# Synergistic effect of antagonists to KRas4B/PDE6 molecular complex in pancreatic cancer

**DOI:** 10.26508/lsa.202302019

**Published:** 2023-10-09

**Authors:** Paola Briseño-Díaz, Michael Schnoor, Martiniano Bello-Ramirez, Jose Correa-Basurto, Arturo Rojo-Domínguez, Leticia Arregui, Libia Vega, Enrique Núñez-González, Luis Andres Palau-Hernández, Carlos Guadalupe Parra-Torres, Óscar Manuel García Córdova, Ernesto Zepeda-Castilla, Eduardo Torices-Escalante, Leticia Domínguez-Camacho, Beatriz Xoconostle-Cazares, Marco Antonio Meraz-Ríos, Sandra Delfín-Azuara, Dayan Andrea Carrión-Estrada, Nicolas Villegas-Sepúlveda, Rosaura Hernández-Rivas, Maria del Rocio Thompson-Bonilla, Miguel Vargas

**Affiliations:** 1 Department of Molecular Biomedicine, Center for Research and Advanced Studies of the National Polytechnic Institute (CINVESTAV-IPN), México City, Mexico; 2 Laboratory of Molecular Modeling and Drug Design of the Higher School of Medicine, National Polytechnic Institute, Mexico City, Mexico; 3 Department of Natural Sciences, Metropolitan Autonomous University, Mexico City, Mexico; 4 Toxicology Department, Center for Research and Advanced Studies of the National Polytechnic Institute (CINVESTAV-IPN), Mexico City, Mexico; 5 Department of Surgical Oncology and General Surgery, Hospital 1 de Octubre, ISSSTE, Mexico City, Mexico; 6 Department of Biotechnology, Center for Research and Advanced Studies of the National Polytechnic Institute (CINVESTAV-IPN), México City, Mexico; 7 Biomedical and Transnational Research, Genomic Medicine Laboratory, Hospital 1 de Octubre, ISSSTE, México City, Mexico

## Abstract

Pancreatic ductal adenocarcinoma (PDAC) has the worst prognosis among all human cancers as it is highly resistant to chemotherapy. K-Ras mutations usually trigger the development and progression of PDAC. We hypothesized that compounds stabilizing the KRas4B/PDE6δ complex could serve as PDAC treatments. Using in silico approaches, we identified the small molecules C14 and P8 that reduced K-Ras activation in primary PDAC cells. Importantly, C14 and P8 significantly prevented tumor growth in patient-derived xenotransplants. Combined treatment with C14 and P8 strongly increased cytotoxicity in PDAC cell lines and primary cultures and showed strong synergistic antineoplastic effects in preclinical murine PDAC models that were superior to conventional therapeutics without causing side effects. Mechanistically, C14 and P8 reduced tumor growth by inhibiting AKT and ERK signaling downstream of K-RAS leading to apoptosis, specifically in PDAC cells. Thus, combined treatment with C14 and P8 may be a superior pharmaceutical strategy to improve the outcome of PDAC.

## Abstract (precis)

C14 and P8 combo reveals potent synergy in PDAC models, promising safer, effective therapy than gemcitabine.

## Introduction

Because it is the most aggressive and chemotherapy-resistant of all human malignancies, pancreatic ductal adenocarcinoma (PDAC) has the worst prognosis. Most PDAC patients survive only 3–6 mo after the first diagnosis, which usually occurs too late because of a lack of symptoms (Kenner et al, 2021). Approximately 99% of PDAC patients have activating mutations in the *KRAS* oncogene, providing PDAC cells with antiapoptotic and chemoresistance properties (Collins et al, 2012). KRas4B-activating mutations substitute residues G12, G13 or Q61, thus inhibiting GTPase-activating protein (GAP)-mediated GTP hydrolysis leading to constitutive activation of KRas4B and downstream signaling pathways such as AKT and ERK (Bryant et al, 2014). DNA synthesis inhibitors such as gemcitabine, 5-fluorouracil (5-FU), and oxaliplatin are first-line treatments for PDAC. However, these drugs have severe side effects, including liver damage, leukopenia, venous collapse, pain, and bone mass loss (Cid-Arregui & Juarez, 2015; Orth et al, 2019). Thus, intensive research is ongoing to find more effective treatment approaches. Uncovering the importance of KRas4B mutations for the initiation, maintenance, and progression of PDAC opened a new field of research to discover and develop inhibitors of KRas4B (Buhrman et al, 2011; Cox et al, 2014). For example, several monoclonal antibodies that target transmembrane tyrosine kinase receptors to decrease KRas4B activation inhibited KRas4B activation in PDAC, albeit with low efficacy (Xu et al, 2017). On the other hand, small organic compounds targeting the KRas4B G12C mutation site have been developed (Wang et al, 2012; Ni et al, 2019), such as the SCH-54292 compound, which binds to the α2 and α3 KRas4B helices and inhibits KRas4B only in cells bearing the G12C mutation (Maurer et al, 2012). Likewise, the GDP analog termed SML-8-73-1 covalently binds to C12 of the G12C-mutated KRas4B, leading to decreased activity (Cox et al, 2014; Hunter et al, 2014; Ni et al, 2019). The compound MRTX84 targets the KRas4B mutation site and inhibits KRas4B^G12C^ mutant tumors in mouse models and patients (Furuse et al, 2018; Hallin et al, 2020; Kim et al, 2020); nevertheless, side effects such as diarrhea, anemia or liver enzyme abnormalities have been reported by the use of these kinds of compounds (Govindan et al, 2019; Wang et al, 2022). A recently discovered compound termed sotorasib or AMG510 targets the KRas4B mutation site inhibiting KRas4BG12C; this disrupts Switch 1/2 (SW1/2) that conforms the P2 pocket of KRas and locks KRas in a GDP-bond state, provoking inhibition of kinase ERK phosphorylation. Evaluation of sotorasib using xenografts models showed significant tumor growth inhibition (Canon et al, 2019; Hong et al, 2020). A significant effect in the clinical evaluation was observed with lung cancer patients. However, the experimental evaluation of sotorasib and adagrasib using Ba/F3 cells transduced with KRas^G12C^ and containing secondary mutations such as Y96D and Y96S showed resistance to both inhibitors (Koga et al, 2021). PDE6δ mediates KRas4B transport from the endoplasmic reticulum to the plasma membrane for its subsequent activation, thus forming the KRas4B/PDE6δ heterodimeric complex in the cytoplasm (Cox et al, 2014; Dharmaiah et al, 2016; Casique-Aguirre et al, 2018). This transport mechanism presents an opportunity to design compounds that stabilize and sequester the heterodimeric complex in the cytoplasm, thus preventing KRas4B activation at the plasma membrane (Cox et al, 2014; Casique-Aguirre et al, 2018; Paola et al, 2020). One such compound is deltarasin, which interacts with PDE6δ with high affinity and prevents recognizing the posttranslational modification present on KRas4B, causing accumulation of KRas4B in the cytosol, thus preventing KRas4B activation and tumor progression (Zimmermann et al, 2013). Unfortunately, deltarasin showed high cytotoxicity in nonmalignant pancreatic duct cells, even at low concentrations (Casique-Aguirre et al, 2018; Paola et al, 2020). Thus, deltarasin analogs, termed deltasonamides, have been developed and have shown a higher cytotoxic effect on PDAC cells at lower concentrations, although they have also exhibited cytotoxicity in normal cells (Martin-Gago et al, 2017; Chen et al, 2019). Consequently, we proposed a novel therapeutic strategy involving the design of compounds that stabilize the complex instead of preventing its formation (Casique-Aguirre et al, 2018; Cruz-Nova et al, 2018). Such compounds could stabilize and sequester the KRas4B/PDE6δ heterodimeric complex in the cytoplasm, thus preventing KRas4B activation at the plasma membrane (Cox et al, 2014; Dharmaiah et al, 2016; Paola et al, 2020). In addition, our research team identified several unique compounds with potent anticancer activities against colon cancer cells (Cruz-Nova et al, 2018, 2022) and pancreatic cancer cells (Casique-Aguirre et al, 2018; Paola et al, 2020), disrupting the KRas signaling pathway and producing specific cell death via apoptosis. The structural basis of the stabilization and sequestration of the KRas4B/PDE6δ heterodimeric complex, as proposed by us, has recently been identified (Yelland et al, 2022). This study confirmed our previous data that KRas4B/PDE6δ stabilization inhibits KRas-mediated ERK signaling (Casique-Aguirre et al, 2018). The proposed complex-stabilization hypothesis has also been applied to a different target, namely 14-3-3ζ proteins and the estrogen receptor alpha (ERα) (Gigante et al, 2020). Further analyzing our database of 38 potential compounds that can stabilize the KRas4B/PDE6δ heterodimeric complex (Casique-Aguirre et al, 2018), we found that compound C14 showed even higher cytotoxic antitumor activity than compounds D14 and C22, which is likely because of subtle chemical modifications in their primary structure compared with C14. In addition, it is important to point out that the identification and evaluation of new compounds analogous to the lead compound is important because these normally have a higher antineoplastic pharmacological potency than the one shown by the lead compound. This information might influence the development of more effective and innovative PDAC treatment programs. Here, we show compound C14 and its analog P8 exhibit the highest specific cytotoxic activity in preclinical PDAC models. Moreover, we demonstrate that combining C14 and P8 has specific synergistic antitumor effects in vivo compared with single treatments. Importantly, C14 and P8 do not show adverse side effects in mice. Thus, we propose C14 and P8 as novel treatment strategies for PDAC patients that may result in better survival, superior to treatments such as gemcitabine.

## Results

### Compound C14 has a higher interaction energy on K-Ras4BG12D/PDE6δ and K-Ras4BG12D/PDE6δ complex targets and causes a higher decrease in cell viability in pancreatic cancer cell lines

**Figure 1.**
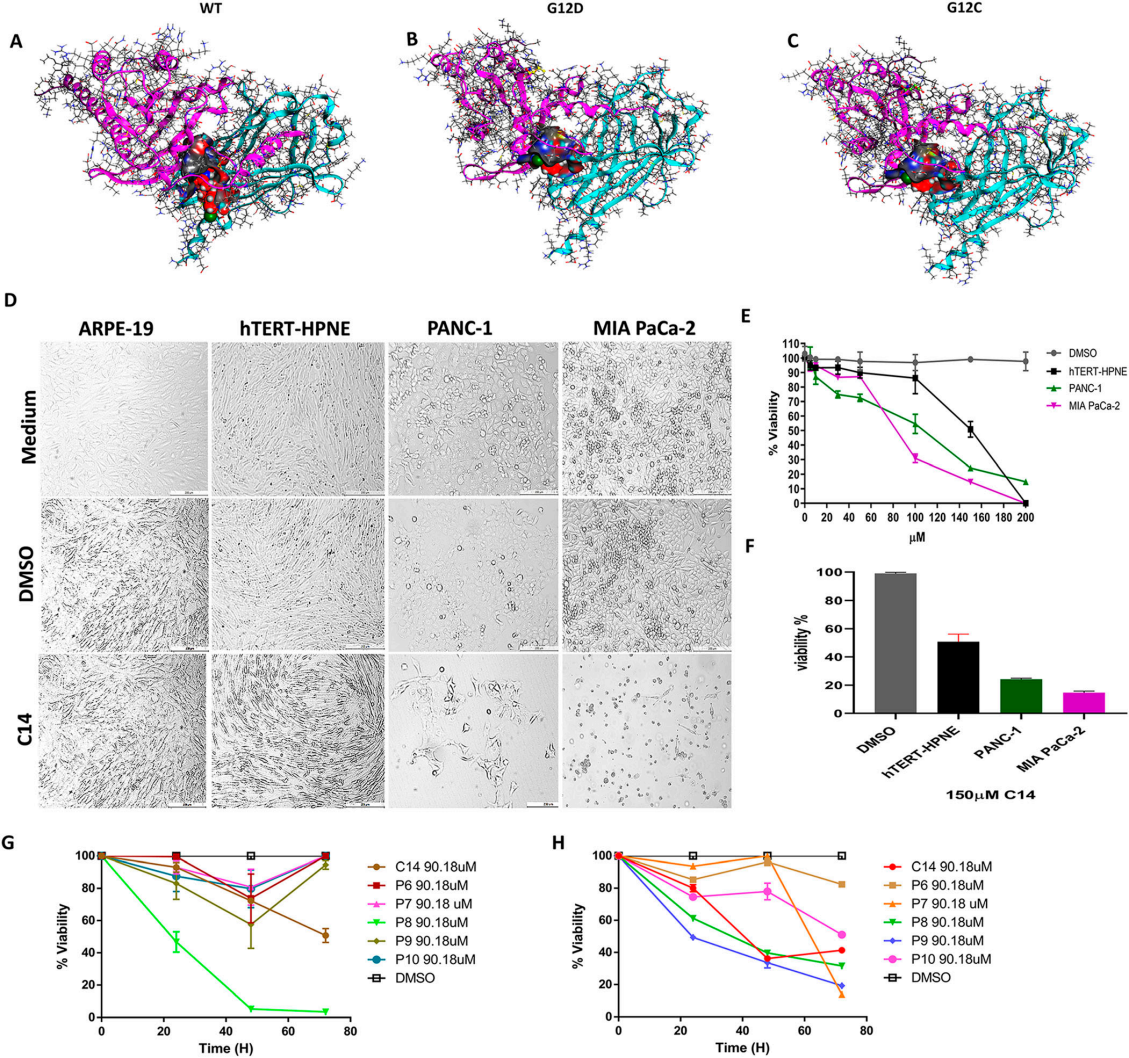
C14 stabilizes the K-Ras4B/PDEδ complex and inhibits the growth of human pancreatic cancer cell lines. **(A)** Interaction of compound C14 (red) with the K-Ras4B^wt^ (purple) PDE6δ (aqua) complex. **(B)** Interaction of compound C14 with the K-Ras4B^G12D^/PBDE6δ complex. **(C)** Interaction of compound C14 with the K-Ras4B^G12C^/PBDE6δ complex. **(D)** Representative bright-field images of the cell lines ARPE-19, hTERT-HPNE, PANC-1, and MIA PaCa-2 treated with 100 μM C14, DMSO as vehicle and untreated control cells. Bar = 20 μm. **(E)** Dose response of MIA PaCa-2, PanC-1 pancreatic ductal adenocarcinoma (PDAC) cells and the normal pancreatic cell line hTERT-HPNE treated with different concentrations of C14 compound for 72 h and compared with relative control DMSO. **(F)** Percentage of PDAC cell lines viability at 72 h treated with 150 μM of the C14 compound. **(G, H)** Relative cell viability curves after treatment of the PDAC cell line MIA PaCa-2 (G) and the normal pancreatic cell line hTERT-HPNE (H) with different compounds known to target the K-Ras4B/PBDE6δ complex were compared with negative control DMSO. (n = 5), with a significance of *P* < 0.001.

To know the molecular recognition sites and to determine the degree of affinity of compound C14 a small organic molecule with a molecular weight of 344.8 g/mol (Tables S1 and S2) on the different versions of the heterodimeric complex molecules target such as KRas4B^WT^/PDE6δ, KRas4BG12D/PDE6δ, and KRas4BG12C/PDE6δ, molecular analysis was carried out as previously described in the see the Materials and Methods section. To accomplish this goal, it was first necessary to measure and analyze the coupling and interaction energies of compound C14 with the heterocomplexes KRas4BWT/PDE6, KRas4BG12D/PDE6, and KRas4BG12C/PDE6 (Fig 1A–C upper panel). In comparison to the non-mutated form KRas4BWT/PDE6, compound C14 had a higher affinity for the molecular complexes KRas4BG12D/PDE6 and KRas4BG12C/PDE6, according to the results of molecular dynamics (MD) simulations coupled to the MMGBSA approach (Gbind −86.60; Gbing −100.30; and Gbind −79.63, respectively) (Table S2). We carried out viability assays to determine the cytotoxic effect of C14 using the PDAC cell lines PANC-1 and MIA PaCa-2 because of the high affinity of the K-Ras4BG12D/PDE6 and KRas4BG12C/PDE6 complexes for C14 and the significance of these complexes in pancreatic cancer maintenance and progression. In these cell lines, the KRas4G12D mutation, which is the most prevalent in PDAC, is present. Because normal pancreatic duct cell line hTERT-HPNE is a non-cancerous cell, we employed it as a control. Given the high affinity of the K-Ras4B^G12D^/PDE6δ and KRas4B^G12C^/PDE6δ complexes for C14 and the importance of these complexes in pancreatic cancer maintenance and progression, we performed viability assays to measure the cytotoxic effect of C14, using the PDAC cell lines PANC-1 and MIA PaCa-2, because these cell lines present the KRas4G12D mutation which has the highest frequency in PDAC, the normal pancreatic duct cell line hTERT-HPNE al cells were also used as cytotoxic specificity controls, because they are non-cancerous cells. Bright-field microscopy analysis of cell lines treated with 100 μM of C14 for 72 h showed a robust cytotoxic effect of C14 in PANC-1 and MIA PaCa-2 cell lines. Still, no significant damage over hTERT-HPNE was observed in their growth and morphology (Fig 1D, middle panel). Cell viability was further analyzed by measuring cellular ATP levels after treatment with different concentrations of C14. MIA PaCa-2 and PANC-1 cells were sensitive to treatment in a dose-dependent fashion with half-maximal inhibitory concentrations (IC_50_) of 90.18 and 103.5 μM, respectively (Fig 1E and F middle panel). Of note, C14 did not show significant cytotoxic activity up to 100 μM in noncancerous hTERT-HPNE cells. However, higher concentrations also induce cytotoxicity with an IC_50_ of 171.4 μM. These results suggest that C14 has a strong and specific cytotoxic activity against PDAC cells, which are well known to present this type of mutation at the KRAS.

Table S1. Interactions of compound C14 and P8 on the K-Ras4B/PDE6δ complex.

Table S2. Binding free energy components of protein–protein and protein–ligand complexes (in kcal/mol units).

### Analog P8 exhibits improved antineoplastic properties than lead compound C14

**Figure S1.**
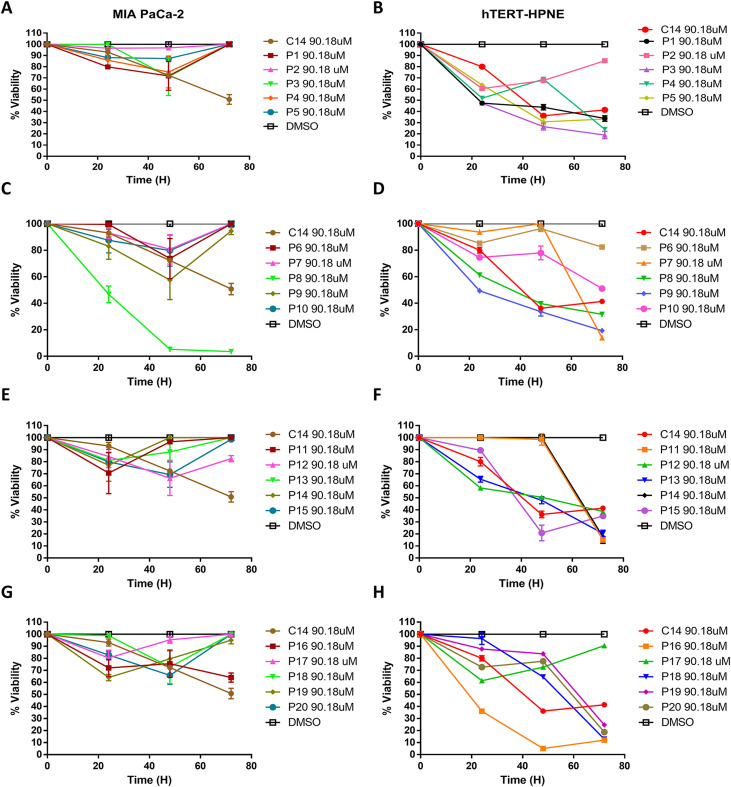
Evaluation and identification of analogs of compound C14. **(A, B)** Evaluation of cell viability after treatment with P1, P2, P3, P4, and P5 analogs of C14 at 90.18 μM in MIA PaCa-2 (A) and hTERT-HPNE (B) cells. **(C, D)** Evaluation of cell viability after treatment with P6, P7, P8, P9, and P10 analogs of C14 at 90.18 μM in MIA PaCa-2 (C) and hTERT-HPNE (D) cells. **(E, F)** Evaluation of cell viability after treatment with P11, P12, P13, P14, and P15 analogs of C14 at 90.18 μM in MIA PaCa-2 (E) and hTERT-HPNE (F). **(E, F, G, H)** Evaluation of cell viability after treatment with P16, P17, P18, P19, and P20 analogs of C14 at 90.18 μM in MIA PaCa-2 (G) and hTERT-HPNE (H) n = 6; ***P < 0.001.

A search for analogs was conducted using the basic structure (pharmacophore) from the leading compound C14, a database from the Enamine library in Kyiv, Ukraine (www.enamine.net), and the Molecular Operating Environment (MOE) 2014.09 software to identify a compound with improved antineoplastic properties compared with that of the leading compound C14. Identification of C14 compound’s pharmacophore was carried out using structure–activity relationship and quantitative structure–activity relationship analysis. After careful examination and acquisition of the docking results (Fischer et al, 2021), we were able to detect 335 potentials analogs with 80–90% similarity to the pharmacophore from the C14 compound; however, only 20 C14 analogs were selected (Table S3) because those meet the Lipinski’s rules (Benet et al, 2016) such as a molecular weight less than 500 kD, less than five hydrogen bond donors, and acceptors, and less than five rotational bonds, therefore having higher target specificity and being able to pass freely through the plasma membrane. The identified analogs showed up to 20% structural changes compared with the lead compound C14. All 20 C14 analogs (Table S3) retained the acetamide and chromene moieties, and the structural modifications to the lead compound were as follows: the addition of amino groups, benzenes, pyridines, and nonaromatic heterocycles that increased its interaction with the KRas4B/PDE6δ molecular complex (Table S3). Using C14 IC50 (90.18 M) as a reference, MIA PaCa-2 cells were exposed to all analogs for varying amounts of time. After 24, 48, and 72 h, ATP luminescence was used to determine which analogs of the central compound C14 had the most significant cytotoxic effect (Fig S1A–H). Only compound P8 showed a significant cytotoxic effect on MIA PaCa-2 cells after 24 h, significantly more potent than C14 (Fig S1G lower left panel). P8 did not kill normal pancreatic cells hTERT-HPNE at this concentration (Fig S1H). Despite showing 80% or more similarity with the pharmacophore of C14, all other tested compounds did not show strong and specific cytotoxic effects in MIA PaCa-2 cells (Fig 1C; lower right panel; Fig S1E–H). Therefore, they were not further analyzed. In conclusion, we identified a new analog compound called P8 with enhanced biochemical properties to bind to K-Ras4BG12D/PDE6δ and KRas4BG12C/PDE6δ mutant heterocomplexes, which also showed better antineoplastic activity than the one leading the C14 compound. However, a number of compounds, including P1; P2; P3; P4, P7, P8, P9, and P10, did not exhibit antineoplastic activity against the MIA PaCa-2 cell line and additionally showed a cytotoxic impact on the normal pancreatic cell line hTERT-HPNE (Fig S1A–D), and as a result, were excluded from this analysis.

Table S3. Analogs to the leader compound C14 selected by means of bioinformatics programs.

### P8 has higher interaction energy and a more potent cytotoxic effect on PDAC cell lines with KRas4B mutations than the lead compound C14

**Figure 2.**
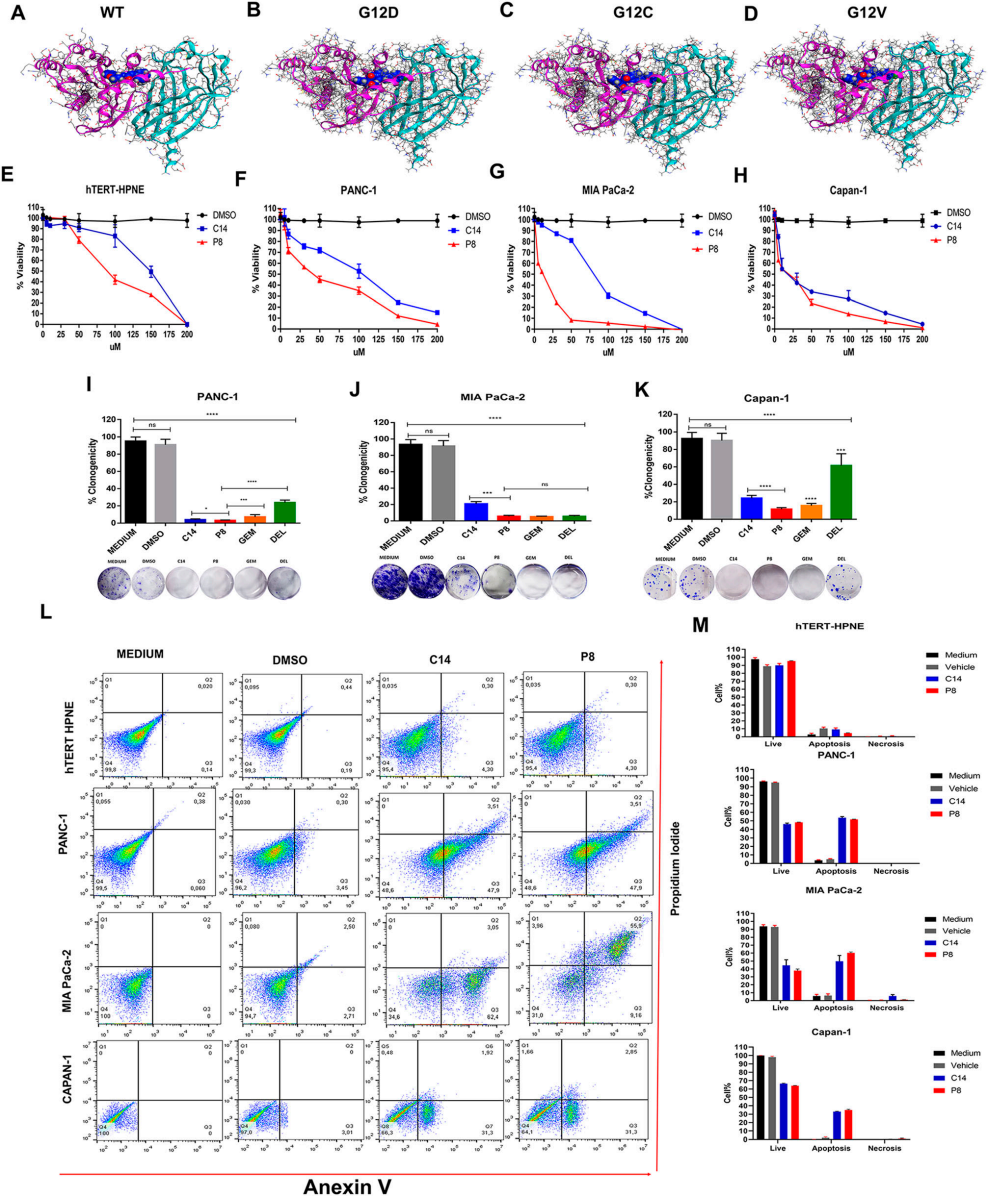
P8 analog stabilizes the K-Ras4B/PDEδ complex and inhibits growth of pancreatic ductal adenocarcinoma (PDAC) cell lines and induced apoptosis better than C14 compound. **(A)** Interaction of compound P8 (blue) with K-Ras4B^wt^ (pink)/PDE6δ (aqua complex). **(B)** Interaction of P8 with the K-Ras4B^G12D^/PBDE6δ complex. **(C)** Interaction of P8 with the K-Ras4B^G12C^/PBDE6δ complex. **(D)** Interaction of P8 with the K-Ras4B^G12V^/PBDE6δ complex. **(E, F, G, H)** Relative cell viability of the PDAC cells PANC-1, MIA PaCa-2, and Capan-1 and the normal pancreatic cell line hTERT-HPNE treated with different concentrations of P8 for 72 h (n = 5). **(I, J, K)** Clonogenic assays of the PDAC cell lines PANC-1, MIA PaCa-2, and Capan-1 treated with the IC_50_ of P8, C14, gemcitabine, and deltarasin (n = 5). **(L, M)** Cell death analyses by flow cytometry in hTERT-HPNE, PANC-1, MIA PaCa-2, and Capan-1 cells treated with the IC_50_ of P8 and C14, DMSO as vehicle or medium alone after staining with Anexin-V, 7-AAD, and CytoCalcein Violet. **(L, M)** Quantification of the plots shown in (L) n = 5; ***P < 0.001.

After discovering the P8 analog, we further explored its molecular targets, mechanisms, and anticancer properties. To do this, we first conducted a molecular analysis using molecular dynamics to determine the molecular and biochemical properties of P8. We then performed a molecular coupling analysis on the molecules that target the KRas4BWT/PDE6, KRas4BG12D/PDE6, and KRas4BG12C/P (Fig 2A–D). This provided information on the intermolecular contact sites of the different mutated versions of K-Ras4B/PDE6δ heterocomplexes (Table S1), and the parameters of interaction energy of compound P8 on KRas4B^G12D^/PDE6δ, KRas4B^G12C^/PDE6δ, and KRas4B^G12V^/PDE6δ complex targets (Table S2). We obtained the interaction energy values of ΔG −101.77, ΔG −103.35, and ΔG −126.14 kcal/mol, respectively, for each molecular complex. The results showed that the interaction energy of compound P8 was higher than compound C14 because compound P8 can establish more contact interaction sites on its KRas4B/PDE6δ heterodimeric complex target.

Given the high affinity of the P8 compound for KRas4B/PDE6δ mutant heterocomplexes, we decided to evaluate the impact of this compound on the cell viability of PDAC cells such as PANC-1, MIA PaCa-2, Capan-1, and hTERT-HPNE cells used as a specificity control. The results of the cytotoxicity assays showed that the P8 compound had a higher cytotoxic effect than its lead compound C14 in all PDAC (Fig 2E–H) because the IC_50_ values for P8 were 51.18 μM in PANC-1 cells (Fig 2F), 24.18 μM in MIA PaCa-2 cells (Fig 2G), and 28.96 μM in Capan-1 cells (Fig 2H), all those values were much lower than those of C14 compound. Notably, the IC_50_ value in hTERT-HPNE normal pancreatic cells was 103.45 μM (Fig 2E) and thus much higher than those for PDAC cells showing that the cytotoxic effect of P8 is very selective for PDAC cells. Two of the cellular characteristics of cancerous pancreatic cells are their fast proliferation and chemoresistance. In this sense, clonogenic assays were performed in the presence or absence of C14, P8, gemcitabine (common PDAC chemotherapeutic), and deltarasin (PDE6δ inhibitor) to determine the chemoresistance of the PDAC cell lines (Fig 2I–K). The results showed that C14 and P8 compounds significantly affected the clonogenicity properties of PANC-1 (Fig 2I), MIA Paca-2 (Fig 2J), and Capan-1 (Fig 2K) cells. P8 had the highest and most significant clonogenicity-reducing effect in all three cell lines, whereas deltarasin and gemcitabine compounds had a similar impact only in MIA PaCa-2 cells; Capan-1 and PANC-1 cell lines, and their antineoplastic properties were significantly lower than that of the P8 compound. On the other hand, the assays to determine the type of cell death caused by C14 and P8 compounds showed that both promoted apoptosis in MIA PaCa-2, PANC-1, and Capan-1 cells. However, the P8 compound did so at a much lower IC50 concentration. Therefore, the P8 compound had better pharmacological activity than the C14 compound (Fig 2L and M). Of note, C14 and P8 did not induce significant apoptosis in normal hTERT-HPNE cells. The number of necrotic cells was neglectable in all conditions. In summary, at low concentrations, P8 showed the highest cytotoxic, clonogenicity-decreasing, and pro-apoptotic effects.

### C14 and P8 inhibit K-Ras, ERK, and AKT activation in PDAC cell lines

**Figure 3.**
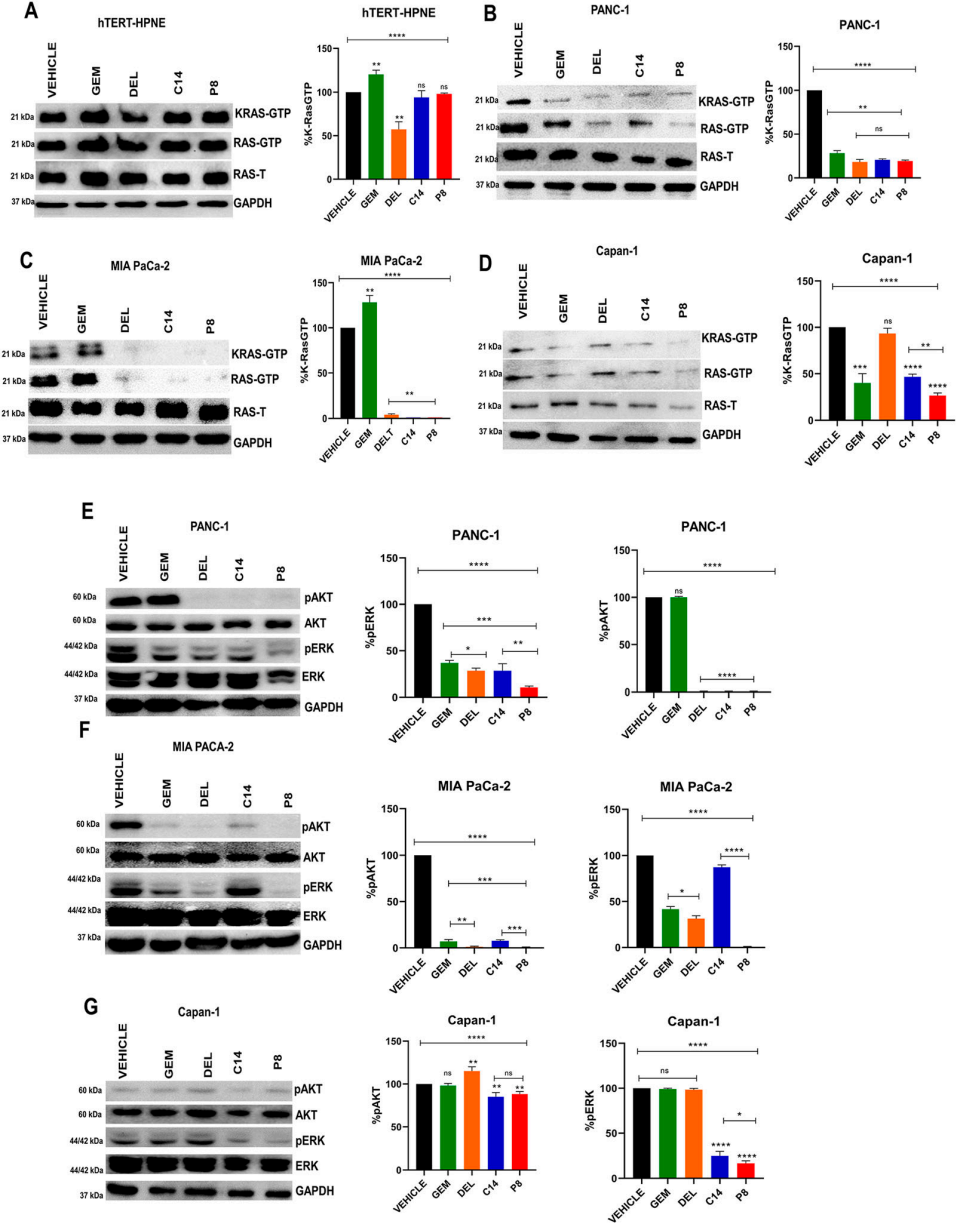
P8 and C14 decrease the activation of K-Ras and phosphorylation of AKT and ERK in pancreatic ductal adenocarcinoma cell lines with K-Ras mutation. **(A, B, C, D)** K-Ras-GTP, K-Ras-GDP expression determined by Western blot in hTERT-HPNE (A), PANC-1 (B), MIA PaCa-2 (C), and Capan-1 (D) cell lines treated with the IC_50_ of P8, C14, gemcitabine, and deltarasin for 3 h. Total protein extracts were precipitated using RAF-RBD beads. Total RAS (Ras-T) and GAPDH are shown as loading controls. Pixel intensities of K-Ras GTP were normalized to total RAS and GAPDH. **(E, F, G)** pAKT, AKT, pERK, ERK expression determined by Western blot in PANC-1 (E), MIA PaCa-2 (F), and Capan-1 (G) cell lines treated with the IC_50_ of P8, C14, gemcitabine, and deltarasin or vehicle. The intensity of pAKT, AKT, pERK, ERK relative to GAPDH was determined by densitometric analysis. GAPDH was used as a loading control. Quantification of pixel intensities of pERK and pAKT relative to total ERK and AKT levels, respectively, are shown in the graphs to the right. Data are shown as SDM; n = 5; ***P < 0.001. Source data are available for this figure.

We performed Ras-GTP pull-down assays and Western blots to determine whether C14 and P8 compounds would affect the activation of KRas and KRas-dependent signaling pathways (Fig 3A–D). The results of these assays showed that there is a potent inhibition of KRas activation in PANC-1 (harboring the G12D mutation) (Fig 3B), MIA PaCa-2 (carrying the G12C mutation) (Fig 3C), and Capan-1 cells (harboring the G12V mutation) (Fig 3D) (these mutations are the three most common KRas mutations in PDAC). However, neither C14 nor P8 chemicals reduced KRas activation in hTERT-HPNE normal cells, which were employed as a control for healthy cells (Fig 3A). This decreased KRas activation on KRas-dependent signaling pathways like ERK and AKT were then examined. In all PDAC cell lines treated with chemicals C14 and P8, we discovered decreased phosphorylation levels and consequent activation of both AKT and ERK. Interestingly, the results varied according to the mutation of each PDAC cell line. AKT activation decreased by 100% in PANC-1 cells treated with C14 or P8 and MIA PaCa-2 cells treated with P8. Compound P8 decreased ERK activation from 80 to 99% in PANC-1 and Capan-1 cells (Fig 3E–G). Thus, C14 and P8 significantly inhibit KRas signaling pathways in PDAC cell lines with different oncogenic addictions.

### Characterization of primary cultures from PDAC

**Figure S2.**
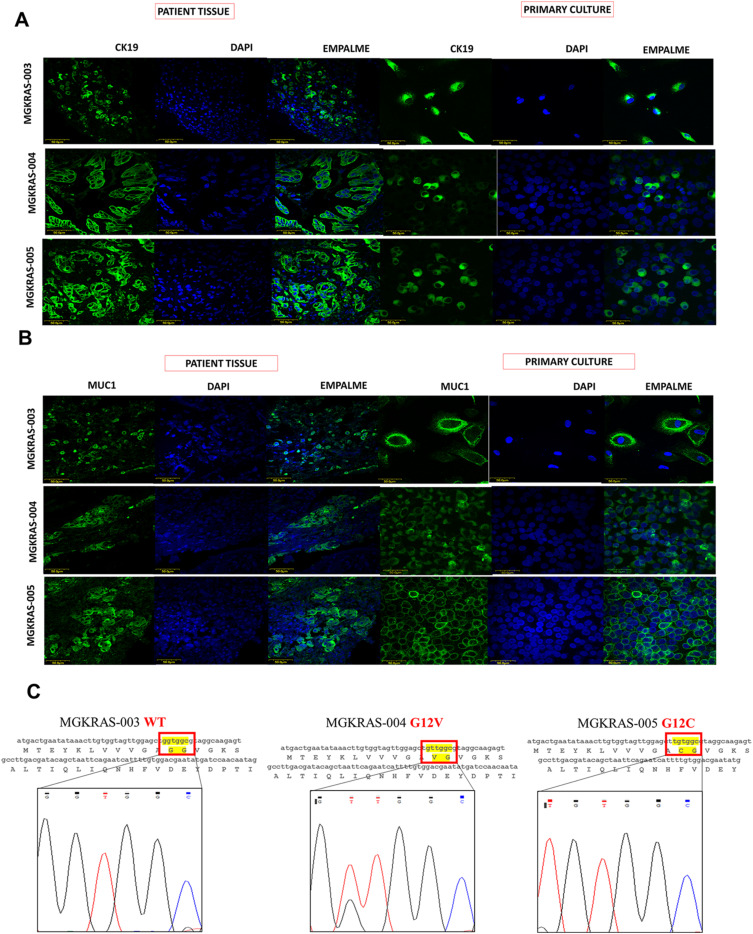
Characterization of pancreatic ductal adenocarcinoma (PDAC) tissues and primary cells. **(A)** Expression of CK19 in MGKRAS003, MGKRAS004, and MGKRAS005 PDAC tissues and primary cells by confocal immunofluorescence microscopy (Leica SP8, Barcelona, Spain). Bar = 50 µm; n = 3. **(B)** Expression of MUC1 in MGKRAS003, MGKRAS004, and MGKRAS005 PDAC tissues and primary cells by confocal immunofluorescence microscopy. Bar = 50 µm; n = 3. **(C)** Nucleotide sequences and histograms of sequencing of exon 2 containing KRAS in MGKRAS003, MGKRAS004, and MGKRAS005 tissues and primary cells. Source data are available for this figure.

**Figure S3.**
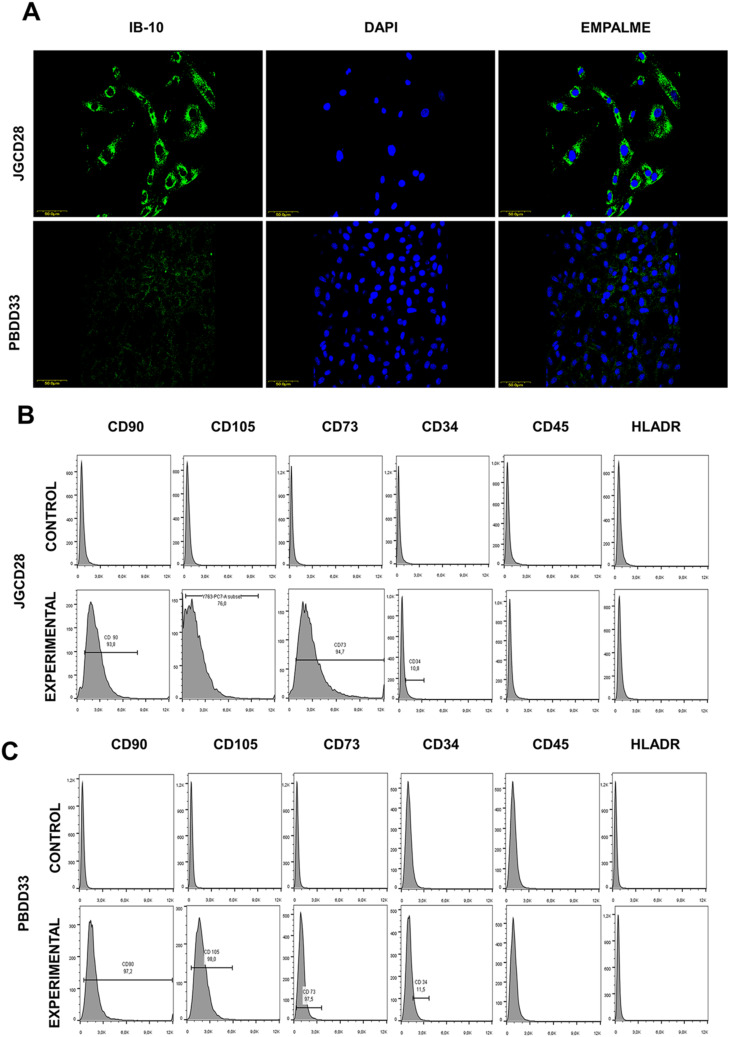
Characterization of skin-derived primary cell cultures. **(A)** Expression of IB-10 in PBD033 and JGC028 primary skin-derived cultures by confocal immunofluorescence microscopy (Leica SP8, Barcelona, Spain). Bar = 50 µm; n = 3. **(B, C)** Expression of CD90, CD105, CD73, CD34, CD45, and HLADR in PBD033 and JGC028 primary skin-derived cultures as analyzed by flow cytometry after correction for autofluorescence.

We obtained 20 pancreatic cancer samples from patients aged 40–100 yr, with most patients being women between 40–59 yr (Table S4). These data contrast with other reports worldwide, which have found the highest incidence in men aged 60–80 yr (He et al, 2013). Samples of normal epithelial tissue were obtained from healthy patients (PBDD33 and JGCD28) as controls. Three fresh PDAC tissue samples were used to establish primary cultures of pancreatic cancer cells termed MGKRAS003, MGKRAS004, and MGKRAS005. Two normal tissues were used to establish primary cultures of epithelial cells that were passaged five times. Tissues and primary cells were analyzed for the presence of neoplastic ductal cells using CK19 as a marker (Fig S2A upper panel). CK 19 was present in both samples, indicating our primary cultures consisted of ductal cells. Another marker often used in clinical practice to identify cancer cells is CA19-9/MUC1 (Fig S2B middle panel). MUC1 was expressed in patient-derived PDAC tissues and primary PDAC cultures, indicating that our primary cultures consisted mainly of neoplastic tissue-derived ductal cells. Next, we analyzed other markers such as CK 7, CK 19, CEA, MUC1, MUC4, MUC16, EGFR, vimentin, cytoplasmic β-catenin, E-cadherin, and Ki-67 (Table S4). Next, using PCR and sequencing exon 2 of the *kras* gene, we identified *kras* mutations in two primary cultures of pancreatic cancer (Fig S2C lower panel), namely MGKRAS004 G12V and MGKRAS005 G12C, whereas the tissue MGKRAS003 expressed WT *kras*. The G12V mutation is reported as the most chemoresistant in PDAC (Fig S2C). We performed the same characterization in primary cultures from normal epithelial tissue observing the presence of epithelial and mesenchymal markers in only one of the samples. We also performed an exhaustive characterization of epithelial tissue fibroblasts using immunofluorescence markers such as IB-10 and cytometry markers such as CD90, CD105, CD73, CD34; CD45, and HLA-DR where we identified epithelial tissue-derived mesenchymal cells in the PBDD33 sample and fibroblasts in the JGCD28 sample (Fig S3A–C). Consequently, our primary cultures of pancreatic cancer cells belonged to stage 4, thus supporting the diagnosis of highly infiltrating and invasive PDAC.

Table S4. Characterization of biomarkers of origin and malignancy in primary cultures.

### Primary PDAC cell cultures are more sensitive to C14 and P8 than to conventional chemotherapeutics

**Figure 4.**
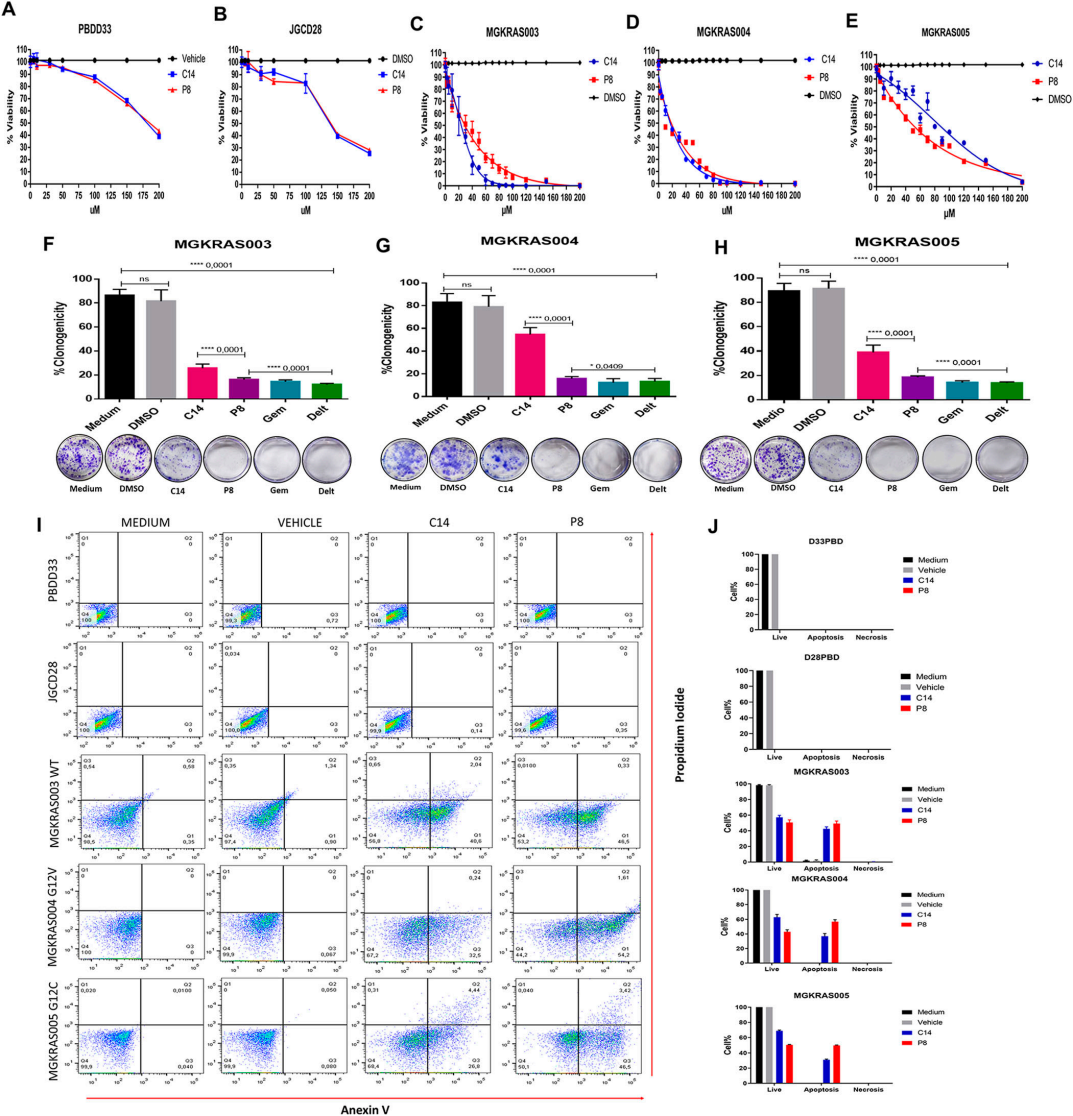
P8 and C14 inhibit the growth of and induce apoptosis in primary pancreatic ductal adenocarcinoma (PDAC) cell cultures. **(A, B, C, D, E)** Effects of P8 and C14 at various concentrations (5, 10, 30, 50, 100, 150, and 200 μM) for 72 h in the primary noncancerous cultures PBD033 and JGC028, and the primary PDAC cultures MGKRAS003, MGKRAS004, and MGKRAS005. **(F, G, H)** Clonogenic assays of the primary PDAC cultures MGKRAS003, MGKRAS004, and MGKRAS005 treated with the IC_50_ of P8, C14, gemcitabine, and deltarasin. **(I, J)** Cell death analyses of PBD033, JGC028 MGKRAS003, MGKRAS004, and MGKRAS005 were determined by flow cytometry after staining with annexin-V, 7-AAD, and CytoCalcein Violet. **(I, J)** Quantification of the plots is shown in (I). Data are shown as SDM; n = 5; ***P < 0.001.

**Figure S4.**
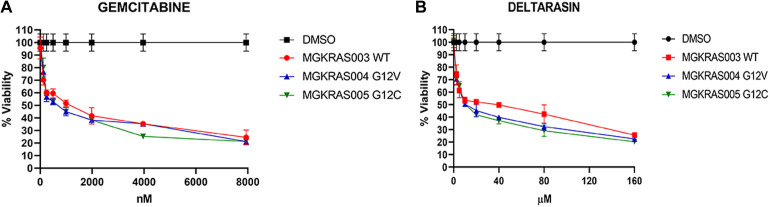
Evaluation of viability of primary cultures treated with gemcitabine and deltarasin. **(A)** Effect of gemcitabine on cell viability at various concentrations (0–8,000 nM) after treatment for 72 h in the primary pancreatic ductal adenocarcinoma cultures MGKRAS003 WT, MGKRAS004G12V, and MGKRAS005G12C. **(B)** Effect of deltarasin at various concentrations (0–8,000 nM) after treatment for 72 h on cell viability in the primary pancreatic ductal adenocarcinoma cultures MGKRAS003 WT; MGKRAS004G12V and MGKRAS005G12C (n = 3); ***P < 0.001.

After characterizing the primary pancreatic cancer cell cultures and control cells, we performed viability assays by measuring cellular ATP levels in primary cultures after 3 d of treatment. We identified IC_50_ values of 15.8 μM for C14 in MGKRAS003, 18.3 μM in MGKRAS004, and 118.9 μM in MGKRAS005 (Fig 4A–E upper panel). In the normal epithelial cells PBDD33 and JGCD28, we found IC_50_ values of 189 μM and 130 μM, respectively. For P8, we detected IC_50_ concentrations of 22.3 μM in MGKRAS003, 18.03 μM in MGKRAS004, 37.5 μM in MGKRAS005, 192 μM in PBDD33, and 145 μM in JGCD28 (Fig 4A–E). Thus, C14 and P8 have a specific cytotoxic effect on primary PDAC cultures and only showed cytotoxicity in nonmalignant primary cultures at very high concentrations. These data also showed that primary PDAC cultures were more sensitive to compounds C14 and P8 than conventional therapeutics. We evaluated the cytotoxic effects of gemcitabine and deltarasin on primary PDAC cell cultures MGKRAS003WT, MGKRAS004G12V, and MGKRAS005G12C, obtaining IC__50__ concentrations of 1,000 nM for gemcitabine and 10 μM for deltarasin, which were twice as high as the reported concentrations for these compounds on pancreatic cancer cell lines (Fig S4A and B). Next, we evaluated the clonogenicity of MGKRAS003, MGKRAS004, and MGKRAS005 using C14, P8, gemcitabine, and deltarasin (Fig 4F–H, middle panel). Here, we observed a significantly more substantial decrease in cell growth in all three primary PDAC cultures treated with P8 than in C14. Similar to the data observed in PDAC cell lines, P8 induced apoptosis in the primary PDAC cells MGKRAS003, MGKRAS004, and MGKRAS005, at lower IC_50_ concentrations compared with C14 (Fig 4I and J lower panel). P8 did not induce apoptosis in the nonmalignant primary cells PBDD33 and JGCD28. Thus, P8, compared with C14, showed more potent cytotoxicity, decreased clonogenicity, and pro-apoptotic effects in primary PDAC cells at even lower concentrations.

### C14 and P8 decrease the activation of K-Ras and downstream signaling pathways from primary PDAC cells

**Figure 5.**
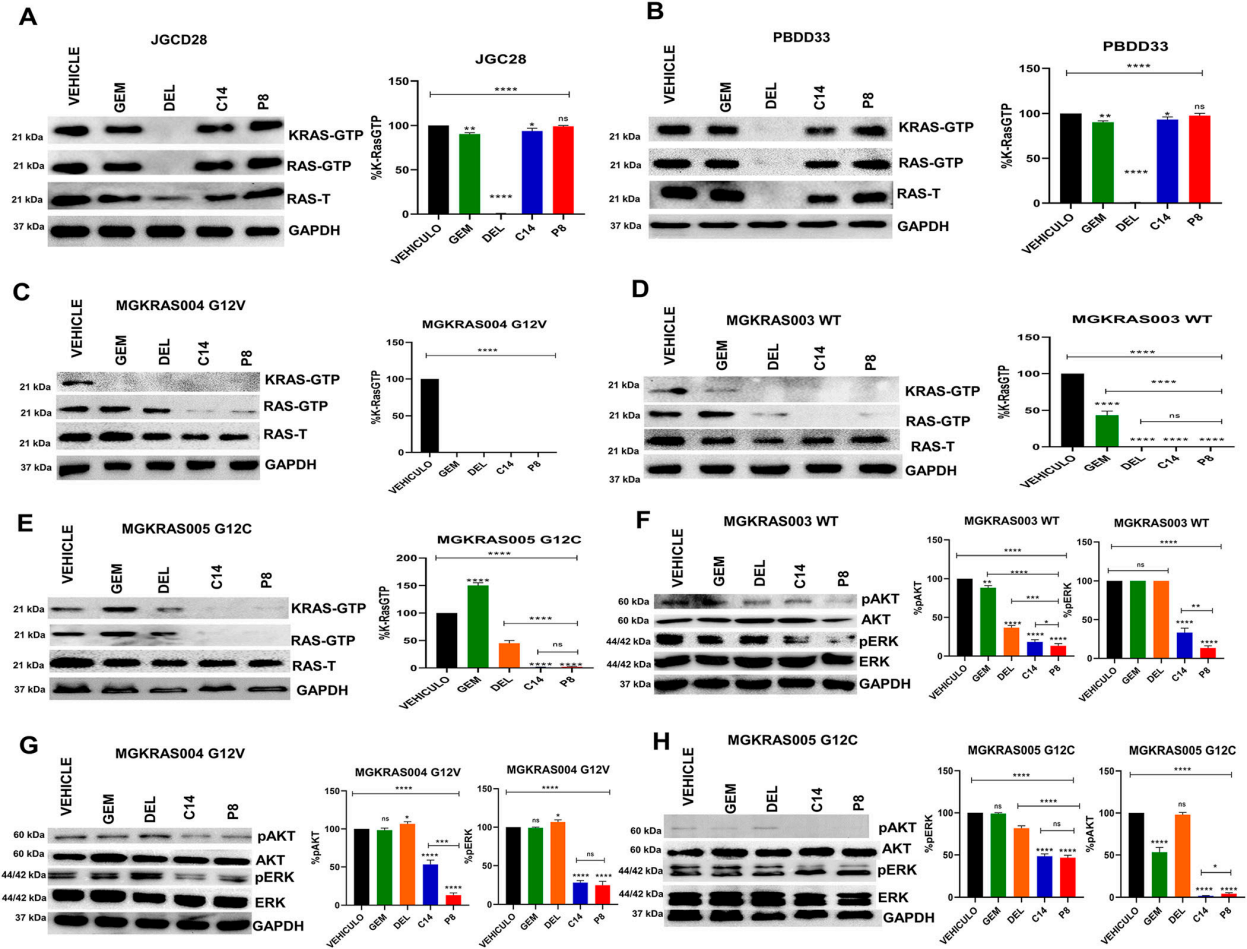
P8 and C14 decrease the activation of K-Ras and phosphorylation of AKT and ERK in primary pancreatic ductal adenocarcinoma cell cultures with K-Ras mutation. **(A, B, C, D, E)** K-Ras-GTP, K-Ras-GDP expression determined by Western blot in PBD033 (A), JGC028 (B), MGKRAS003 (C), MGKRAS004 (D), and MGKRAS005 (E) cells treated with the IC_50_ of P8, C14, gemcitabine, and deltarasin for 3 h. Total protein extracts were precipitated using RAF-RBD beads. Total RAS (Ras-T) and GAPDH are shown as loading controls. Pixel intensities of K-Ras GTP were normalized to the controls. **(F, G, H)** pAKT, AKT, pERK, ERK expression determined by Western blot in MGKRAS003 (F), MGKRAS004 (G), and MGKRAS005 (H) cells treated with the IC_50_ of P8, C14 compounds, and compared with negative control DMSO (vehicle) and positive controls gemcitabine and deltarasin. The intensity of pAKT, AKT, pERK, ERK relative to GAPDH was determined by densitometric analysis. GAPDH was used as a loading control. Quantification of pixel intensities of pERK and pAKT relative to total ERK and AKT levels, respectively, are shown in the graphs to the right. Data are shown as SDM; n = 5; ***P < 0.001. Source data are available for this figure.

To determine whether our compounds would affect the activation of KRas and KRas-dependent signaling pathways in primary PDAC cells, we also performed Ras-GTP pull-down assays using gemcitabine, deltarasin, C14, and P8 (Fig 5A–E). C14 and P8, unlike deltarasin, did not decrease KRas activation in the nonmalignant primary cultures PBDD33 (Fig 5B) and JGCD28 (Fig 5A). Of note, C14 and P8 decreased K-Ras activation by more than 90% in the primary PDAC cell cultures MGKRAS003 (Fig 5C), MGKRAS004 (Fig 5D), and MGKRAS005 (Fig 5E). Next, we performed Western blot assays to measure AKT and ERK phosphorylation to investigate whether this decrease in KRas activation would impact KRas-dependent signaling. Indeed, we observed a decreased activation of AKT and ERK in the primary PDAC cultures MGKRAS003 (Fig 5F), MGKRAS004 (Fig 5G), and MGKRAS005 (Fig 5H) treated with C14 or P8. Results varied depending on the mutation present in each primary pancreatic cancer cell culture. We observed an 80% decrease in AKT activation in MGKRAS003 and MGKRAS004 cells treated with compound P8 and a 95% decrease in AKT activation in MGKRAS005 cells treated with C14 or P8. In addition, P8 decreased ERK activation by 50–80% in MGKRAS003, MGKRAS004, and MGKRAS005, indicating that C14 and P8 significantly inhibit KRas-dependent signaling pathways in primary pancreatic cancer cell cultures. On the other hand, deltarasin induces phosphorylation of AKT and ERK in MGKRAS004, which presents the *kras*^^G12V^^ mutation. Deltarasin decreased AKT phosphorylation by only 50% in MGKRAS003 and decreased ERK phosphorylation by only 15% in MGKRAS005, again showing that C14 and P8 have superior inhibitory effects on KRas activation and its downstream signaling.

### C14 and P8 have synergistic effects on pancreatic cancer cell lines and primary PDAC cultures

**Figure 6.**
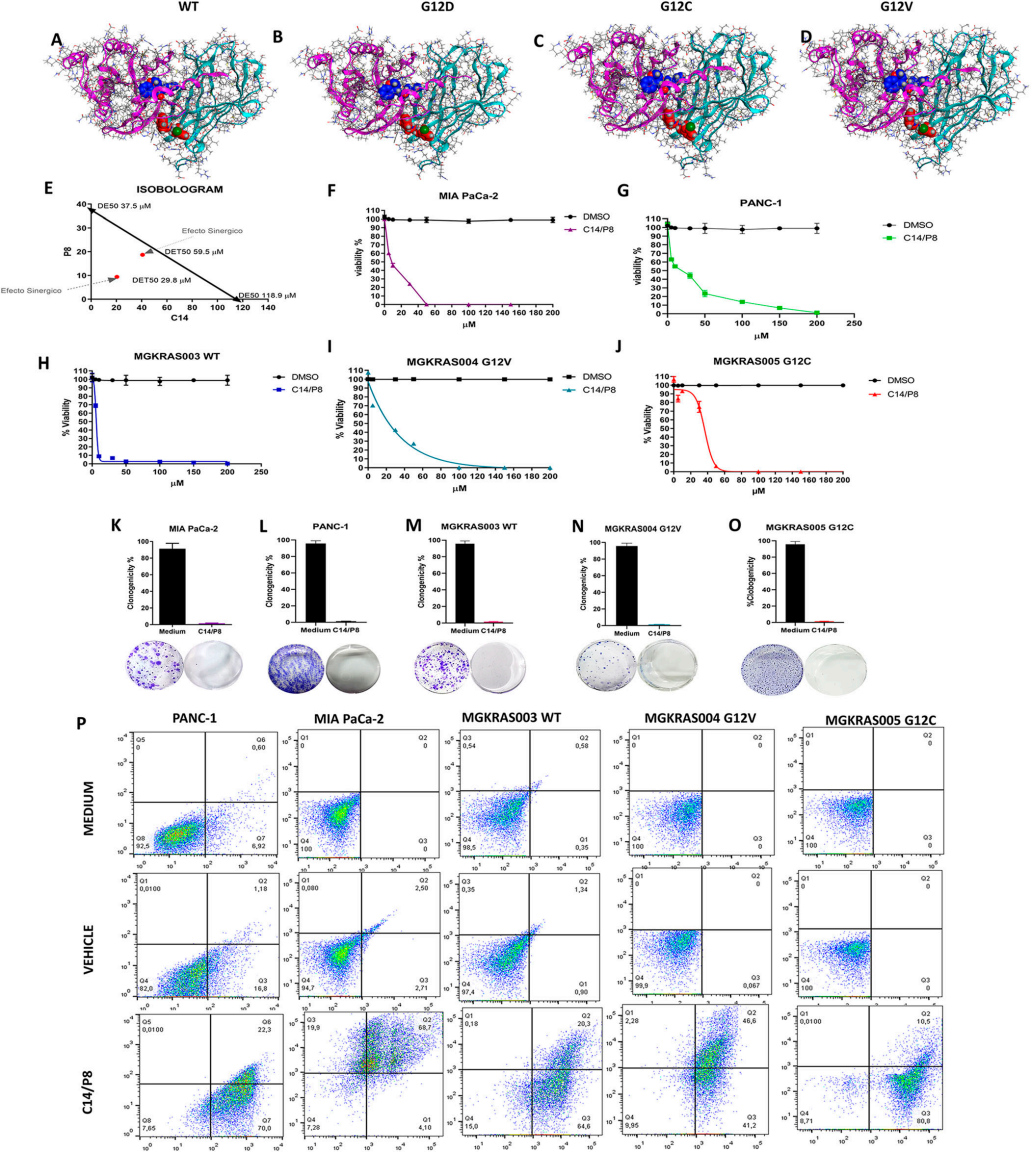
Synergistic effect of P8 and C14 on cell lines and primary pancreatic ductal adenocarcinoma cultures. **(A)** Synergistic interaction of P8 (blue), C14 (red) with the K-Ras4B^wt^ (pink and yellow)/PDE6δ (aqua complex). **(B)** Synergistic interaction of P8/C14 with the K-Ras4B^G12D^/PBDE6δ complex. **(C)** Synergistic interaction of P8/C14 with the K-Ras4B^G12C^/PBDE6δ complex. **(D)** Synergistic interaction of P8/C14 with the K-Ras4B^G12V^/PBDE6δ complex. **(E)** Isobologram of the IC_50_ of compounds P8 and C14. **(F, G, H, I, J)** Dose response on cell viability of MIA PaCa-2 (F) and PANC-1 (G) cell lines and MGKRAS003 (H), MGKRAS004 (I) and MGKRAS005 (J) primary pancreatic ductal adenocarcinoma cultures of compounds P8/C14 at various concentrations of each (5, 10, 30, 50, 100, 150, and 200 μM). **(K, L, M, N, O)** Clonogenic assays of MIA PaCa-2 (K), PANC-1 (L), MGKRAS003 (M), MGKRAS004 (N), and MGKRAS005 (O) treated with the IC_50_ concentration of P8 and C14 compounds. **(P)** Cell death analyses of PANC-1, MIA PaCa-2, MGKRAS003, MGKRAS004, and MGKRAS005 by flow cytometry after staining with annexin-V, 7-AAD, and CytoCalcein Violet. Data are shown as SDM; n = 5; ***P < 0.001.

One of the objectives we set out to evaluate in this work was to study and understand the antineoplastic synergistic properties of both compounds C14 and P8. In this sense, it was important to know the anchoring sites of both compounds on their target molecules. To achieve this, an in silico analysis of compounds C14 and P8 on the different heterodimeric complexes, namely KRas4B^WT^/PDE6δ, KRas4B^G12D^/PDE6δ, KRas4B^G12C^/PDE6δ, and KRas4B^G12V^/PDE6δ, was carried out. The results of this molecular analysis allowed the detection of a differential molecular behavior of their anchoring sites to their target molecules for both compounds. Therefore, we performed docking (Fig 6A–D upper panel) and molecular dynamics investigations of both compounds on the WT and mutant heterodimers (Table S2). These data showed a ΔG_bind_ of −120.77 for KRas4B^WT^/PDE6δ, −153.75 for KRas4B^G12D^/PDE6δ, −186.14 for KRas4B^G12C^/PDE6δ, and −175.59 for KRas4B^G12V^/PDE6δ. With both compounds, the interaction energy was higher with mutant complexes than with WT, suggesting that compounds C14 and P8 may have a synergistic effect when applied together. To assay the synergistic effects of C14 and P8, we performed an isobologram analysis to identify any synergistic, additive or antagonistic effect by plotting the IC_50_ concentrations of both compounds (Fig 6E). We obtained several theoretical data points in the synergistic effect region following the Chou and Talalay method (Chou, 2010). On the other hand, the evaluation of the combined antineoplastic properties of compounds C14 and P8 compounds on pancreatic cancer cell lines and in primary cultures allowed determining the following IC50 values: IC_50_ values of 10.2 μM for MIA PaCa-2 (Fig 6F), 18 μM for PANC-1 (Fig 6G), 5.8 μM for MGKRAS003 (Fig 6H), 8.3 μM for MGKRAS004 (Fig 6I), and 18.9 μM for MGKRAS005 (Fig 6J). These data show that the combination of both C14 and P8 is more effective at much lower concentrations. Using these IC_50_ concentrations of C14 and P8 in combination, we evaluated the clonogenicity of cell lines, and primary cultures found decreases of more than 99% in the clonogenicity of MIA PaCa-2 (Fig 6K), PANC-1 (Fig 6L), MGKRAS003 (Fig 6M), MGKRAS004 (Fig 6N), and MGKRAS005 (Fig 6O). Next, we performed cell death analyses by flow cytometry in PANC-1, MIA PaCa-2, MGKRAS003, MGKRAS004, and MGKRAS005 cells (Fig 6P). C14 and P8, in combination, induced apoptosis in over 90% of cells in the PDAC cell lines and primary PDAC cultures. In summary, these data show strong synergistic effects of C14 and P8 on both PDAC cell lines and primary cultures with different KRas mutations.

### Compounds C14 and P8 have no side effects in mice, unlike gemcitabine

**Figure S5.**
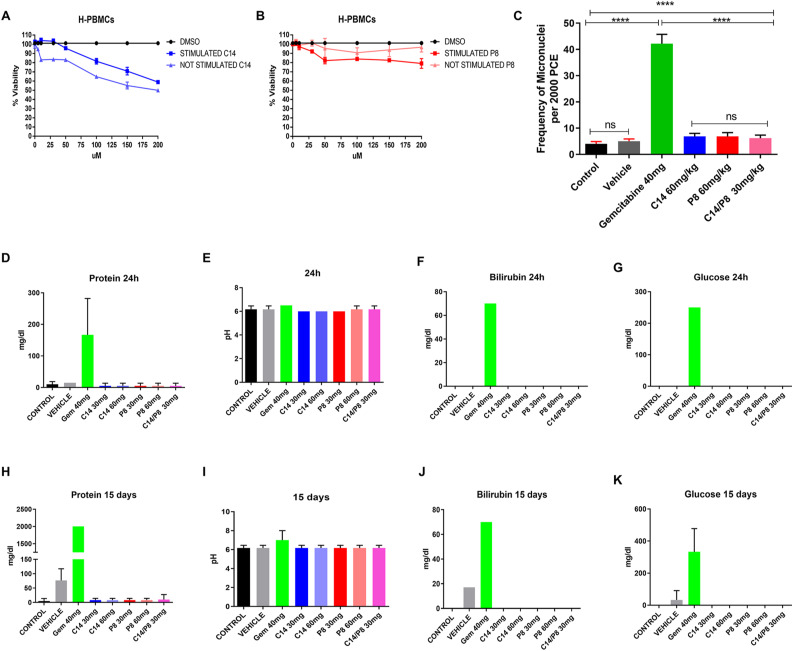
Evaluation of cytotoxicity and genotoxicity of P8 and C14. **(A, B)** Effects of P8 and C14 at different concentrations (5, 10, 30, 50, 100, 150, and 200 μM) on cell viability of stimulated and non-stimulated H-PBMC’s (n = 5). **(C)** Frequency of micronuclei in polychromatic erythrocytes isolated from bone marrow of BALB/c mice treated with 60 μM P8, C14, and 40 μM gemcitabine for 24 h. **(D, E, F, G)** Evaluation of protein presence (D), pH (E), bilirubin (F), and glucose (G) in the urine of BALB/c mice after treatment with 60 μM P8, C14, and gemcitabine for 24 h. **(H, I, J, K)** Evaluation of protein presence (H), pH (I), bilirubin (J), and glucose (K) in the urine of BALB/c mice after treatment with 60 μM P8, C14, and gemcitabine for 16 d; (n = 6); ***P < 0.001.

We used activated, and nonactivated human peripheral blood mononuclear cells (PBMCs) treated with various concentrations of compounds C14 and P8 to conduct a cytotoxicity assay to ensure that our compounds have no harmful effects on nonmalignant cells. The results showed that the IC50 values for C14 in stimulated and non-stimulated PBMCs were 418.3 M and 443 M, respectively, and for P8 in stimulated and non-stimulated PBMCs were 1,144 μM and 1,371, respectively (Fig S5A and B). These concentrations exceeded 100 times the concentrations obtained in PDAC primary cultures. They were 4–10 times higher than those in nonmalignant cell lines and primary cultures, thus demonstrating the specificity of compounds C14 and P8 for PDAC cells and primary cultures. To evaluate the genotoxicity of C14 and P8 in BALB/c mice, we administered once at 24 h, 30 or 60 mg/kg of C14 or P8, a combination of 30 mg/kg C14 and P8, 40 mg/kg gemcitabine, or vehicle (0.05% carboxymethyl cellulose in PBS containing 0.5% DMSO), and analyzed micronuclei in the bone marrow. We found similar levels of micronuclei in around 5% of bone marrow cells of untreated mice and mice treated with C14 and P8 alone (Fig S5C). However, over 40% of bone marrow cells contained micronuclei in mice treated with gemcitabine, indicating that compounds C14 and P8, unlike gemcitabine, do not induce genotoxicity in the mouse bone marrow. BALB/c mice received intraperitoneal injections of C14 and P8 once for 24 h or once daily for 15 d to assess their side effects in vivo (Fig S5H–K). Controls included untreated mice, doses of 30 or 60 mg/kg of C14 or P8, the combination of 30 mg/kg C14 and P8 and 40 mg/kg gemcitabine, or vehicle (0.05% carboxymethyl cellulose in PBS with 0.5% DMSO) (Fig S5D–G). Mice were euthanized, and urine was collected to measure protein, pH, bilirubin, and glucose. After 24 h treatment, we found increases to 300 mg/dl protein (Fig S5D), 70 mg/dl bilirubin (Fig S5F), and 250 mg/dl glucose (Fig S5G) in mice treated with gemcitabine. In contrast to gemcitabine treatment, no changes compared with controls were detected in mice treated with C14, P8, or the C14/P8 combination, indicating that these compounds had no severe side effects in mice. Similar data were obtained after an extended treatment over 15 d. Several side effects were observed in BALB/c mice treated with gemcitabine (Table S5), including diarrhea, rectal prolapse, intestinal torsion, decreased muscle mass, weight, and appetite loss. By contrast, mice treated with C14, P8, or the combination showed no signs. Blood counts revealed leukopenia and neutropenia in mice treated with gemcitabine. The blood chemistry of gemcitabine-treated mice revealed an increase in liver enzymes such as alkaline phosphatase (AP), alanine aminotransferase (ALT), aspartate aminotransferase (AST), glucose 500 mg/dl (Fig S5K), 2,000 mg/dl protein (Fig S5H), and 70 mg/dl bilirubin levels (Fig S5I). Notably, such abnormalities were not found in mice treated with C14, P8 or the C14/P8 combination. The effects of gemcitabine in mice led us to consider whether these same effects occur in pancreatic cancer patients receiving this treatment. By evaluating the medical records of patients who agreed to participate in this study, we found asthenia (G2), nausea (G2), epigastralgia, fatigue (G2), hyporexia (G2), leukopenia (G2), neutropenia (G2), low bone marrow reserve, thrombocytosis, elevated liver enzymes (G3), bone pain, and weight loss (Table S6). Thus, chemotherapy was discontinued and replaced with palliative treatment because of these severe side effects. Our results suggest that compounds C14 and P8 have no side effects and are not genotoxic in murine models, making them excellent candidates for clinical trials for PDAC treatment.

Table S5. Side effects obtained in BALB/c mice treated with gemcitabine, C14, P8, and C14/P8.

Table S6. Side effects presented in patients treated with standard chemotherapy in Mexico.

### The combination of C14 and P8 decreases tumor growth in heterotopic and orthotopic xenograft murine models

**Figure 7.**
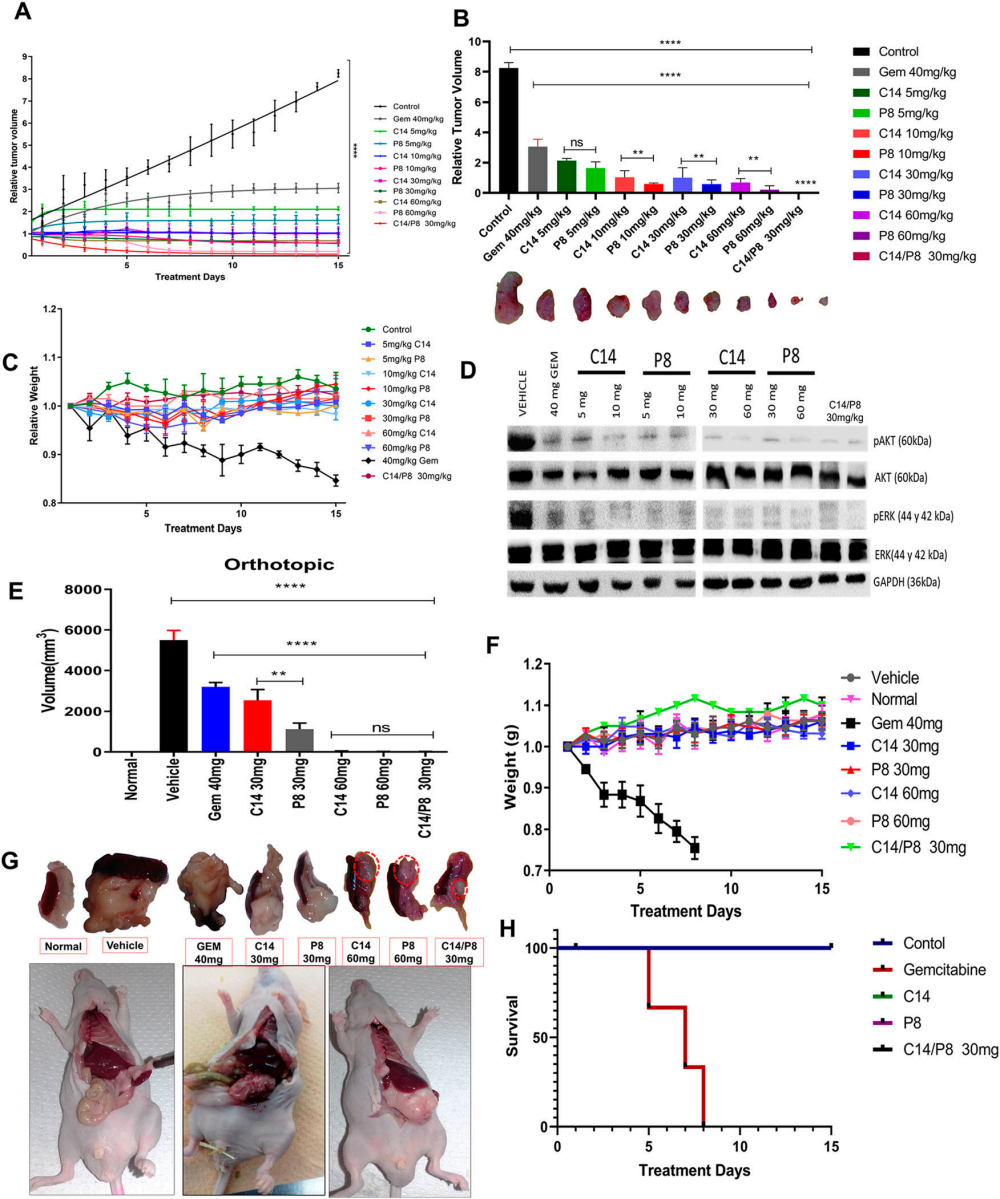
The combination of P8 and C14 reduces tumor growth in subcutaneous and orthotopic xenograft models. **(A)** Effects of P8, C14, and C14/P8 at different concentrations (5, 10, 30, and 60 mg/kg, and the combination of 30 mg/kg) in a subcutaneous xenograft model of injection of MIA PaCa-2 cells in the skin of the back of male Nu/Nu mice (n = 6). **(B)** Quantification of tumor volume every day after treatment with P8, C14, and C14/P8 at different concentrations (n = 6). Below the graph are images of representative tumors of each of the treatments performed. **(C)** Body weight was measured daily during treatment with P8, C14, and C14/P8. **(D)** Representative WB of MIA PaCa-2 tumor lysates treated with P8, C14, gemcitabine shows complete inhibition of AKT and ERK phosphorylation using total AKT, ERK, and GAPDH as loading controls. The relative quantification of the Western blot results is shown in the graphs. **(E)** Effects of P8, C14, and C14/P8 at different concentrations (30 and 60 mg/kg, and the combination of 30 mg/kg) in an orthotopic xenograft model of injection of MIA PaCa-2 cells in the pancreas of mice NU/NU male (n = 6). **(F)** Body weight was measured daily during treatment with P8, C14, and C14/P8. **(G)** Representative images of the effect of treatment with P8, C14, P8/C14, and gemcitabine. **(H)** Survival graph during treatment with P8, C14, P8/C14, and gemcitabine. Data represent mean±SDM of at least six independent experiments; ***P < 0.001. Source data are available for this figure.

**Figure 8.**
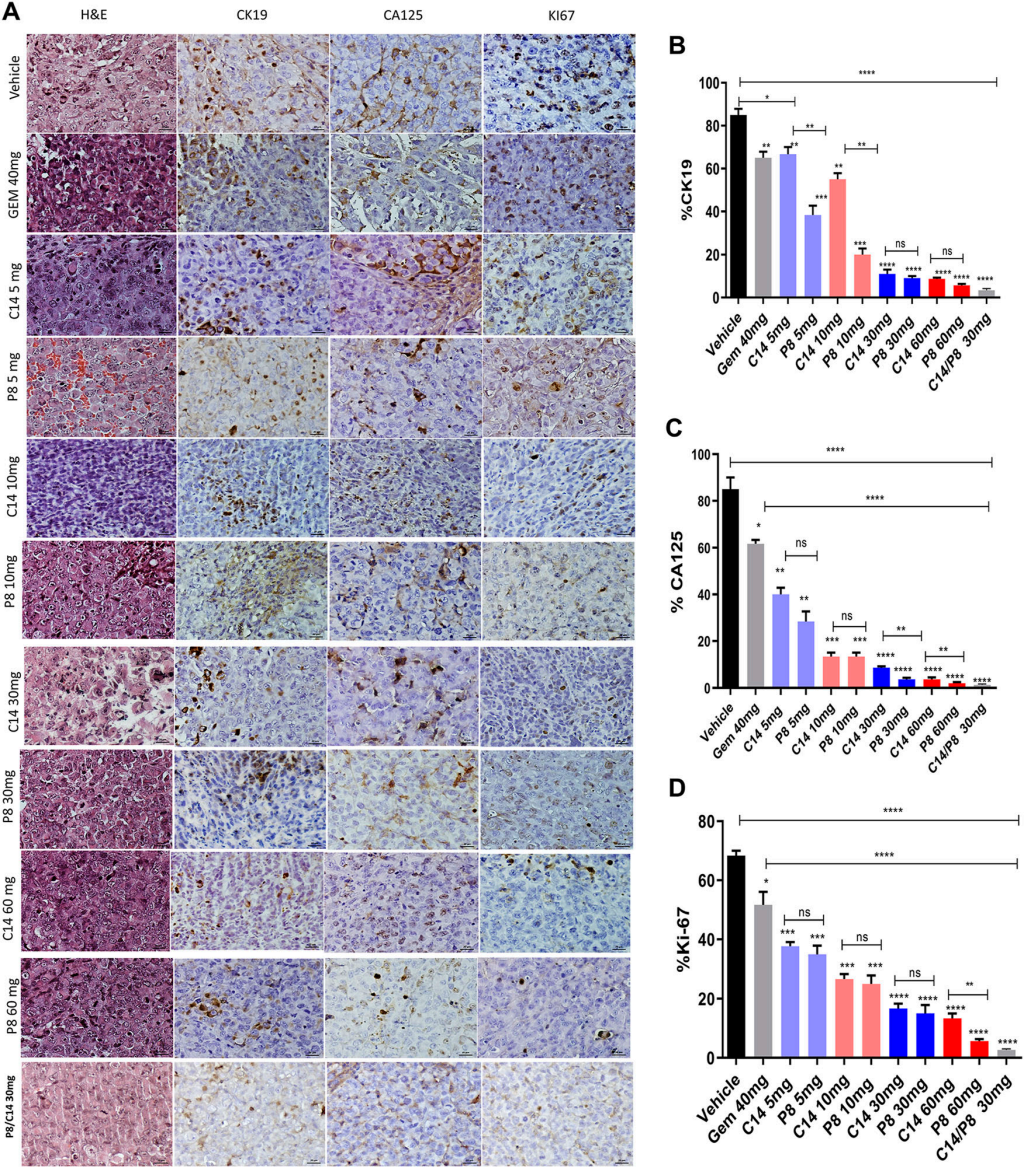
Evaluation of the malignancy markers CK19, CA125, and Ki-67 in tumors derived from subcutaneous xenografts, by means of immunohistochemistry. **(A)** Representative hematoxylin–eosin and immunohistochemistry images of the tumor sections derived from mice treated with P8, C14, P8/C14, gemcitabine or vehicle. **(B, C, D)** Quantification of signal intensities of CK19 (B), CA125 (C), and Ki-67 (D). Tumor sections were counted at 100 cells per field with six fields per section: ***P < 0.001. Source data are available for this figure.

To evaluate the antitumor effect of compounds C14 and P8 in a subcutaneous xenograft model (Fig 7A–D), we injected 5 × 10^6^ MIA PaCa-2 cells into the backs of NU/NU mice. We administered C14 and P8 once daily for 15 d. As controls, mice were treated with 40 mg/kg of gemcitabine once every 3 d for 15 d or vehicle (0.05% carboxymethyl cellulose in PBS with 0.5% DMSO). Our daily measurements of the tumor volume during therapy revealed a significant reduction of more than 50% at all C14 and P8 concentrations and more than 80% at 30 or 60 mg/kg of C14 or P8. The most significant decrease was obtained with 60 mg/kg of P8. Of note, tumor growth decreased by more than 95% with the combination of P8 and C14 at 30 mg/kg (Fig 7B). These effects were superior to those obtained with gemcitabine. We did not observe weight loss either with C14 or P8 alone or with the combination of both (Fig 7C). By contrast, body weight decreased 20% in the gemcitabine group. Next, we analyzed AKT and ERK phosphorylation in tumor tissues after different treatments (Fig 7D). All concentrations of C14 and P8 reduced AKT and ERK phosphorylation. Still, the most potent effect was at 60 mg/kg of C14 and P8 in combination. Because KRas signaling triggers cell proliferation and survival, we measured the proliferation and concentration of cancer cells in tissues after treatment. After showing that compounds C14 and P8 had antitumor effects in the heterotopic model, we evaluated the antitumor effects of compounds C14 and P8 using the orthotopic model. We directly inoculated 1 × 10^6^ MIA PaCa-2 cells into the pancreas of NU/NU mice. 7 d after surgery, different treatments were administered intraperitoneally with vehicle of 30 and 60 mg/kg of C14, 30 and 60 mg/kg of P8, a combination of 30 mg/kg of C14/P8, and 40 mg/kg of gemcitabine (Fig 7E–H). C14 and P8 showed reduced tumor growth in a dose-dependent fashion (Fig 7E). Even better results were observed with the different doses of compound P8, which decreased tumor growth by more than 90%. Of note, the combination of P8 and C14 at 30 mg/kg reduced tumor growth by 95%, suggesting that the two compounds at these concentrations have a strong synergistic effect in the orthotopic model. Gemcitabine-treated mice lost more than 20% of their body weight and had to be euthanized after 8 d (Fig 7F). Representative pictures of the tumors and pancreas for each treatment are shown in Fig 7G. Body weight of mice treated with compounds C14 and P8 did not decrease. In contrast to gemcitabine-treated mice, these groups showed 100% survival at the end of treatment, which showed only 40% after 7 d (Fig 7H). We performed immunohistochemistry for CK 19 as a marker of ductal cells, CA125 (Mucin 16) as a marker of cancer cells, and Ki-67 as a marker of proliferation. Representative images (Fig 8A) showed that the signal of CK 19, CA125, and Ki-67 decreased with increasing concentrations of C14 or P8. We observed a substantial decrease in these markers when applying 30 mg/kg of C14 and P8 in combination and a 70% reduction in proliferating neoplastic ductal cells. When using 60 mg/kg of P8, neoplastic ductal cells were reduced by over 90% (Fig 8B–D). In addition, we observed almost complete prevention of proliferation and survival of cancer cells using the C14/P8 combination at 30 mg/kg, further supporting the synergistic effect of the compounds on PDAC markers. Thus, our data demonstrate that C14 and P8 have superior synergistic antitumor effects compared with gemcitabine in different mouse PDAC models.

### The combination of C14 and P8 reduces tumor growth in primary culture xenograft models

**Figure 9.**
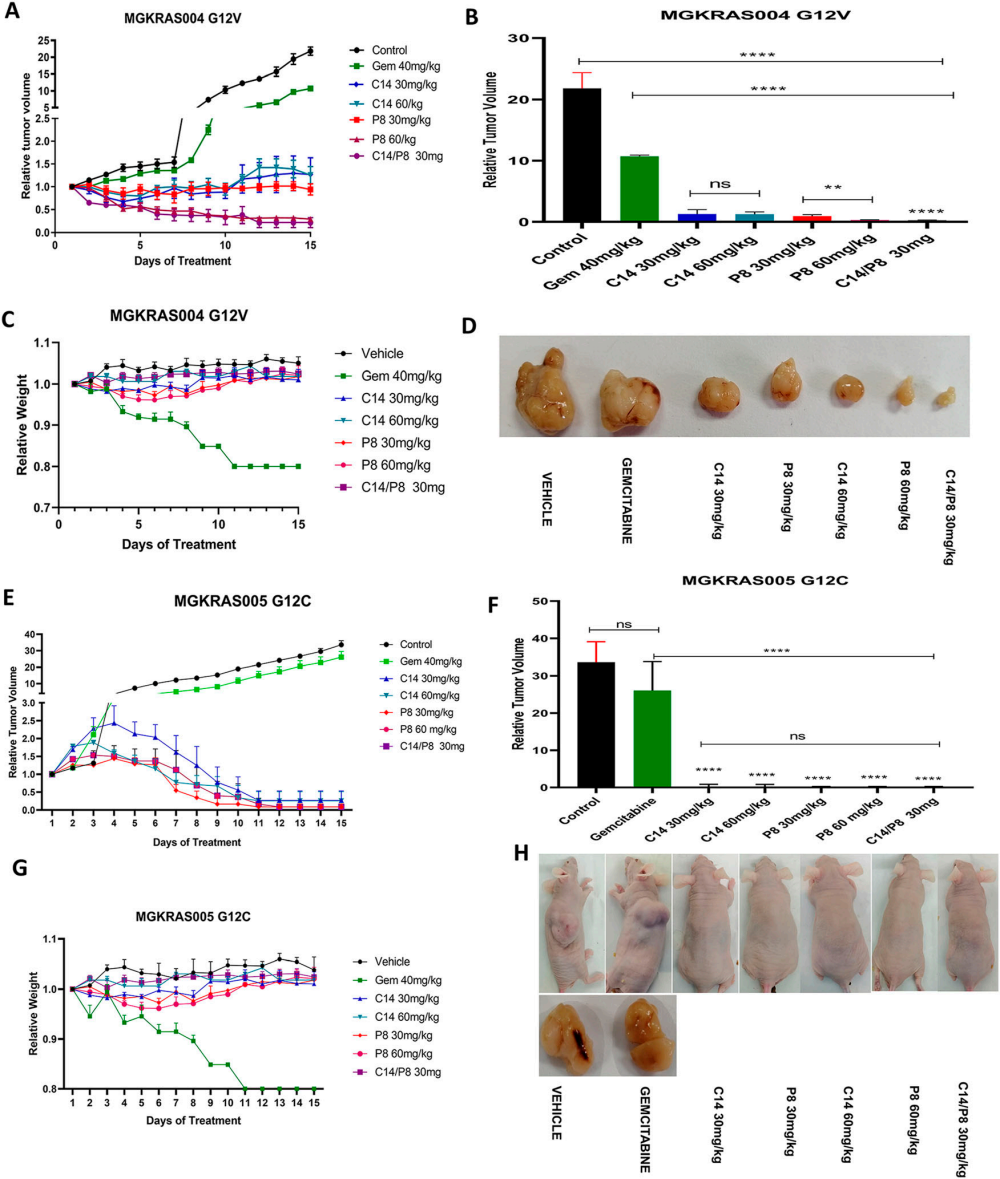
Combined treatment with P8 and C14 reduces tumor growth in primary culture xenograft models. **(A)** Tumor growth effects of P8, C14, and C14/P8 at different concentrations (5, 10, 30, and 60 mg/kg, and a combination of 30 mg/kg each) in a subcutaneous xenograft model using MGKRAS004 cells. **(B)** Effect of tumor growth after treatment with P8, C14, and C14/P8 at different concentrations on the MGKRAS004 xenograft. **(C)** Body weight was measured daily during treatment with P8, C14, and C14/P8. **(D)** Representative images of MGKRAS004 tumors obtained from each group. **(E)** Effects of P8, C14, and C14/P8 at different concentrations (5, 10, 30, and 60 mg/kg, and a combination of 30 mg/kg each) in a subcutaneous xenograft model (n = 6) using MGKRAS005 cells. **(F)** Effect of tumor growth after treatment with P8, C14, and C14/P8 at different concentrations on the MGKRAS005 xenograft. **(G)** Body weight was measured daily during treatment with P8, C14, and C14/P8. **(H)** Mice representative images of MGKRAS005 tumor development obtained from each group of treatments with C14, P8 compounds, and gemcitabine. Data represent mean±SDM of at least six independent experiments; ***P < 0.001.

To evaluate the antitumor effects of both C14 and P8 compounds in subcutaneous xenograft models, the primary cultures MGKRAS004 and MGKRAS005 were used as they harbor the G12V and G12C mutations, which are the third and second most common mutations in pancreatic cancer and are known to be highly resistant to chemotherapy (Hayama et al, 2019). We grafted 5 × 10^6^ MGKRAS004 cells into the back of NU/NU mice, with an initial volume of 150 mm^3^. Next, we administered intraperitoneally different concentrations of C14 and P8 (30 and 60 mg/kg and their combination at 30 mg/kg) once daily for 15 d, 40 mg/kg of gemcitabine once every 3 d for 15 d, and vehicle as a control. We observed a significant decrease in tumor growth with the two concentrations of each C14 or P8 compound. At the doses of 30 and 60 mg/kg of C14, tumor size decreased by more than 90% and more than 95% for P8, the reduction in tumor size was most evident at 60 mg/kg of P8. Of note, with the combination of 30 mg/kg of C14/P8, we observed a 98% decrease in tumor growth (Fig 9A, B, and D). Tumor growth decreased by 50% when the mice were treated with gemcitabine. Representative pictures of these tumors are shown below the graph. Mice did not lose weight when treated with P8, C14 or P8/C14 (Fig 9C). By contrast, we observed weight loss of around 20% in mice treated with gemcitabine. We also evaluated the antitumor effect in MGKRAS005-derived tumors (with the G12C mutation) in the xenograft model. We applied the same approach as described above. We found an impressive gradual decrease in tumor growth over the experimental period of 15 d until the tumors were practically eradicated by all concentrations of C14, P8 or P8/C14 (Fig 9E and F). In contrast, tumors of mice treated with gemcitabine did not show a significant decrease in tumor growth (Fig 9F). Representative images of treated mice and tumors are shown in Fig 9H. Moreover, gemcitabine-treated mice showed more than 20% weight loss on day 11, and so they had to be euthanized (Fig 9G). Single or combined treatments with P8 and C14 did not cause weight loss. In summary, our data highlight the strong potential of P8 and C14 as superior chemotherapeutics in PDAC.

## Discussion

Here, we studied the effects of the small molecules C14 and P8 on the progression of PDAC and showed that they have specific cytotoxic effects on tumor cells at low concentrations without affecting normal cells. Moreover, in FDA-approved in vivo PDAC models, we demonstrated that these drugs do not have any severe side effects. This is of particular importance as one of the significant problems of PDAC treatment nowadays is the lack of specific and compelling chemotherapeutic agents. Drugs discovered in the 1960s, such as 5-FU and gemcitabine, are still the most appropriate therapy for treating various cancers, including PDAC. However, these chemotherapeutic agents act on DNA and protein synthesis in both normal and malignant cells, thus leading to severe adverse effects.

In many cases, chemotherapy must be stopped and changed for palliative therapy (Schlack et al, 2016; Iyikesici, 2020). Mutations significantly influence the initiation, maintenance, and development of PDAC in the KRas, resulting in its constitutive activation (Zeitouni et al, 2016). Therefore, treatment strategies have emerged to suppress KRas activation at the plasma membrane. Deltarasin was the first compound designed to interfere with KRas4B activation in cancer cells. Deltarasin and its analogs block PDE6δ, the protein that transports KRas4B to its activation site at the cell membrane (Zimmermann et al, 2013; Papke et al, 2016). These compounds interact with the hydrophobic pocket of PDE6δ responsible for transporting the GTPase to the plasma membrane by recognizing the farnesyl group present in the Ras GTPase. Once deltarasin interacts with PDE6δ, the latter cannot recognize the farnesyl group present at the carboxyl end of KRas4B, thus blocking its transport to the plasma membrane. KRas4B exerts its function at the cell membrane by activating different cell signaling pathways related to oncogenic processes. One disadvantage of deltarasin is that it can inhibit various cell signaling pathways because KRas4B is not the only target molecule transported by PDE6δ (Zimmermann et al, 2013). A deltarasin analog was reported in 2016, which showed greater specificity and capacity to inhibit PDE6δ, with an IC_50_ of 24 μM, five times lower than the concentration of the lead compound deltarasin (Papke et al, 2016; Paola et al, 2020). Despite significant efforts in identifying new pharmacological strategies to treat pancreatic cancer, no new compounds that specifically suppress PDAC cell viability have been identified yet. Thus, we have searched for small molecules with potential chemotherapeutic properties and identified compound C14 that showed promising, specific antitumor activity in PDAC cells with different KRas4B mutations (Casique-Aguirre et al, 2018). The C14 showed cytotoxic effects on the PDAC cell lines PANC-1 and MIA PaCa-2 at concentrations much lower than those of compounds D14 and C22 that we reported previously (Casique-Aguirre et al, 2018; Paola et al, 2020). Of note, all these compounds target not only one protein but the entire KRas4B/PDE6δ heterodimeric complex, stabilizing and sequestering the complex and inhibiting the transport of KRas4B to the plasma membrane and its activation there. One strategy to improve existing compounds is to change the primary structure to generate analogs (Chuang et al, 2017). In the present study, we modified compound C14 and generated compound P8, with higher cytotoxic properties toward PDAC KRas4B mutant cells at even lower concentrations than the lead compound C14.

Of note, we observed higher cytotoxic effects of these compounds in the cell lines harboring the G12C and G12V mutations, which are more chemoresistant, with no cytotoxic effects on nonmalignant cells. These specific cytotoxic effects of C14 and P8 on PDAC cell lines suggest that they have different permeabilities and IC_50_ depending on the KRas4B mutation. Moreover, in silico analysis revealed that P8 has a higher interaction affinity for mutant complexes than compound C14. The increase in the affinity of compound P8 is because of the presence of a piperazine moiety with two amino groups that increase the interaction surface with the heterodimeric complex making the interaction with WT and mutant KRas4B/PDE6δ complexes more stable. The piperazine moiety in P8 also provides greater solubility, thus facilitating membrane crossing into target cells to promote its cytotoxic effect with a partition coefficient of 3.99 and a solubility constant of −4.4. Compound C14, which does not have piperazine, has a partition coefficient of 3.63 and a solubility coefficient of −4.4. The in silico analysis of the deltazinone on PDE6δ showed a greater interaction affinity with the PDE6δ binding pocket than its lead compound deltarasin because of the elimination of the phenylbenzimidazole moiety, creating greater specificity of deltazinone toward PDE6δ. The in silico data were corroborated using PDE6δ recombinant protein to perform microcalorimetric techniques (Papke et al, 2016). It will be engaging in the future to obtain the affinity values of compounds C14 and P8 by using biochemical methods such as BIACORE or microcalorimetric techniques to get quantitative interaction data. It will also be critical to perform mass spectrometry to identify all potential other targets of C14 and P8 in PDAC cells.

One of the most important findings of our study is the specificity of both P8 and C14 to induce apoptosis in PDAC cells with KRas4B oncogenic addiction but not in normal cells. This is highly relevant because other compounds do not show such specificity for tumor cells. For example, deltarasin showed a more significant cytotoxic effect on normal hTERT-HPNE cells than MIA PaCa-2 cells (Casique-Aguirre et al, 2018). C14 and P8 also specifically decreased K-Ras activation and downstream signaling pathways in PDAC cell lines that depend on the oncogenic addiction on KRas4B mutations without affecting nonmalignant cells. In contrast, deltarasin reduced downstream signaling pathways and KRas activation in both cellular systems.

Consequently, PDAC cells experience decreased cell proliferation, protein synthesis, transcription, and cell cycle activity and increased apoptotic cell death in response to treatment with C14 and P8. These data highlight the potential of C14 and P8 as PDAC treatments. Indeed, we were also able to confirm these promising effects on pancreatic cancer in primary PDAC cells that showed much lower IC_50_ values for C14 and P8 than those observed in the cell lines. Gemcitabine and deltarasin showed much higher IC_50_ concentrations in these primary cultures. C14 and P8 also significantly decreased K-Ras4B activation and signaling pathways in primary PDAC cell cultures without affecting nonmalignant mesenchymal or fibroblast-like primary cultures, thus confirming their specificity toward pancreatic cancer cells. Given these promising results, we believe that C14 and P8 are the first effective chemotherapeutics that do not affect normal cells. While collecting pancreatic cancer samples from patients, we found that PDAC occurs more frequently in women aged 40–60 yr in Mexico. This is consistent with other studies conducted by the Mexican national health services (Gonzalez-Santiago et al, 2017). By contrast, PDAC is most diagnosed in men aged 60–80 yr worldwide (Sung et al, 2021).

Another important finding of our study is the synergistic action of C14 and P8, resulting in lower doses of each compound without losing efficacy, which may lead to further decreased side effects. The combination of compounds C14 and P8 yielded IC_50_ concentrations nine times lower than those of the individual compounds. Combined application of C14 and P8 at such low concentrations specifically induced apoptosis rather than necrosis in more than 90% of PDAC cells. Moreover, the combination of C14 and P8 caused a 99% reduction in the clonogenicity of PDAC cells. The structural basis for KRas4B/PDE6δ complex formation and simultaneous binding of molecules has been recently demonstrated (Yelland et al, 2022). This study confirmed our previous finding that sequestering this complex in the cytosol prevents KRas activation at the plasma membrane and downstream ERK signaling. It will be interesting to reveal in the future the crystal structure of C14 and P8 bound to the KRas4B/PDE6δ complex, which may help further improve drug efficacy.

Drug combinations are common in treating cancer, infections, pain, and various degenerative diseases (Chou, 2010; Hackman et al, 2020). FDA-approved preclinical models for evaluating drugs with potential chemotherapeutic effects include subcutaneous xenografts, orthotopic xenografts, and PDX xenografts in mice (Shi et al, 2020). Such xenotransplantation models also allow observing the influence of niche differences and cell heterogeneity in each model (Kim et al, 2009). C14 and P8 decreased tumor growth in both subcutaneous and orthotopic xenograft models in a dose-dependent manner with none of the adverse effects observed with gemcitabine. Mechanistically, C14 and P8 decreased KRas4B, AKT, and ERK activation in tumors, ultimately leading to apoptosis and tumor shrinkage.

In all xenograft models, the combination of C14 and P8 demonstrated superior anticancer effects, with almost full tumor eradication and no adverse effects, which were not seen with gemcitabine. Similarly, C14 and P8 inhibited tumor growth in pancreatic cancer PDX models. More pharmacokinetic studies in mice are required to assess the therapeutic potential of chemicals C14 and P8. Future research should examine enhanced tissue targeting employing nanoparticles coated with the chemicals to reduce doses further and boost efficacy. In conclusion, the antineoplastic evaluation of C14 and P8 demonstrated their specificity for PDAC cell lines and primary cultures without affecting nonmalignant and primary cultures. These compounds decreased the activation of KRas4B and its downstream effectors AKT and ERK. These antineoplastic activities showed significant synergy when compounds C14 and P8 were applied together; the combination almost wholly eradicated the xenografted PDAC tumors in mice with no adverse effects or genotoxicity. Therefore, we propose combined administration of C14 and P8 as novel treatment strategies for PDAC patients with better safety and efficacy than conventional chemotherapy. Our preclinical data will hopefully pave the way for such clinical trials.

## Materials and Methods

### In silico docking simulations

The chemical structures of C14 and P8 were obtained from the ENAMINE database 3D Diversity set (www.enamine.net). The crystallographic structure of the KRas4B^WT^/PDE6δ system was derived from PDB 5TAR. Mutations for getting the KRas4B^G12D^/PDE6δ, KRas4B^G12C^/PDE6δ, and KRas4B^G12V^/PDE6δ complexes were introduced with the mutagenesis module of PyMOL. Docking studies were performed using AutoDock 4.2.626 (Rizvi et al, 2013) and MOE Dock ([MOE], 2014:09) to evaluate the interactions between KRas4B^WT^/PDE6δ, KRas4B^G12D^/PDE6δ, KRas4B^G12C^/PDE6δ or KRas4B^G12V^/PDE6δ systems with C14 and P8 compounds. The docking calculations were carried out using the recommended standard parameter settings. We evaluated a maximum of 250,000 poses for C14 on the receptor target (crystallographic contacts between KRas4B with PDE6δ). The grid box with 126 × 126 × 126 points was calculated using Autogrid 4.2. (Morris et al, 2008) with a spacing of 0.375 Å by focusing on the crystallized interface of the proteins. Molecular docking with MOE 2014.09 was performed using the matcher function to generate the initial poses. The best 30 results from the London ΔG score were further refined using energy minimization with MMFF94x force field and rescored using Affinity ΔG scoring.

### Molecular dynamics simulations

The molecular dynamics (MD) simulations of the WT and mutated KRas4B/PDE6δ–ligand complexes with the lowest binding scores predicted through docking studies were performed using the AMBER 16 package (Case et al, 2005) and the ff14SB force field (Hawkins et al, 1996). Ligand charges for non-parameterized residues in proteins were determined using the general Amber force field (Forli et al, 2016) and the AM1-BCC level. For the KRas4B/PDE6δ–ligand complexes, a 15-Å rectangular-shaped box of the TIP3P water model (Hawkins et al, 1996; Forli et al, 2016; Sridhar et al, 2017) was employed. Cl^−^ and Na^+^ ions for the protein–ligand system were placed in the model to neutralize the positive or negative charges around the complex at pH 7. Before the MD simulation, the system was minimized through 3,000 steps of steepest descent minimization, followed by 3,000 steps of conjugate gradient minimization. The systems were then heated from 0 to 310 K during 500 picoseconds (ps) of MD while being restrained in their positions by an NVT ensemble. After 600 ps of constant pressure equilibration at 310 k using the SHAKE algorithm (Childers & Daggett, 2018) on hydrogen atoms and Langevin dynamics for temperature control, isothermal, isobaric ensemble (NPT) MD was performed to adjust the solvent density. The equilibration run was followed by 100 ns-long MD simulations without position restraints under periodic boundary conditions using an NPT ensemble at 310 K. The particle mesh Ewald method was used to describe the electrostatic term (Heggen et al, 2010), and a 10-Å cut-off was used for Van der Waals interactions. Temperature and pressure were preserved using the weak coupling algorithm (Berendsen et al, 1984) with coupling constants τT and τP of 1.0 and 0.2 ps, respectively (310 K, 1 atm). The time of the MD simulation was set to 2.0 femtoseconds, and the SHAKE algorithm (Childers & Daggett, 2018) was used to constrain bond lengths at their equilibrium values. The coordinates were saved for analyses every 50 ps. AmberTools14 was used to examine the time dependence of the root mean squared deviation, the radius of gyration (RG), and clustering analysis to identify the most populated conformations during the equilibrated simulation time.

### Calculation of free binding energies

Free binding energies were calculated using the MMGBSA approach (Miller et al, 2012) provided in the AMber16 suite (Case et al, 2005). Five hundred snapshots were chosen at time intervals of 100 ps from the last 50 ns of MD simulation using a concentration of 0.1 M and the Generalized Born (GB) implicit solvent model [34]. The free binding energy of the protein–ligand system was determined as follows:

$$〖\Delta G〗\_\text{bind}=G^{\wedge }\text{complex}-G^{\wedge }\text{receptor}-G^{\wedge }\text{ligand}$$

〖ΔG〗_bind=〖ΔΕ〗_MM+〖ΔG〗_solvation−TΔS

ΔEMM represents the molecular mechanical force field’s total energy, including the electrostatic (ΔEele) and van der Waals (ΔEvdw) interaction energies. ΔG solvation is the free desolvation energy price upon complex formation estimated from the GB implicit model and solvent-accessible surface area calculations yielding ΔGele-sol and ΔGnpol-sol. TΔS is the solute entropy arising from structural changes in the free solutes’ degrees of freedom when forming the protein–ligand complex.

### Reagents

Small organic compounds identified by virtual screening were purchased from ENAMINE (enamine.net). The compounds were dissolved in 1.5% DMSO (catalog No. 276855-1 L; Sigma-Aldrich). Deltarasin (hydrochloride) was purchased from Cayman Chemical (catalog No. 1440898–82-7).

### Selection of analogs of compound C14

A list of 335 compounds from the company ENAMINE was used to choose the compound C14 analogs. The software program MOE from Chemical Computing Group (www.chemcomp.com) was used to examine potential analogs by running energy minimization, structural similarity, and pharmacophore search tests.

### Cell culture

Human pancreatic cancer cell lines MIA PaCa-2, PANC-1, Capan-1, and human pancreatic cell line hTERT-HPNE were obtained from the American Type Culture Collection (ATCC). Cell lines were grown as monolayers in the specific medium suggested by ATCC. The MGKRAS003, MGKRAS004, and MGKRAS005 primary cultures were plated in 96-well plates at a density of 3 × 10^4^ cells/well and grown for 24 h in DMEM containing 80% of FBS (Thermo Fisher Scientific). The normal human dermis-derived mesenchymal PBDD33 and JGCD28 were isolated using the methods previously described (Park et al, 2015) and cultivated in DMEM media containing 1% antibiotics (penicillin/streptomycin) and 10% FBS (Gibco).

### Cell viability

PBMCs, MGKRAS003, MGKRAS004, and MGKRAS005 primary cultures, PANC-1, MIA PaCa-2, Capan-1 cell lines, and hTERT-HPNE were plated in 96-well plates at a density of 3 × 10^4^ cells/well and cultured for 24 h. Cells were exposed to progressively higher concentrations of C14 and P8 over 72 h in a complete medium. Cells were examined using the CellTiter-Glo kit (Promega) following the manufacturer’s instructions to determine viability. 72 h after the treatment, the IC50 concentration of the substances C14 and P8 was established using a curve fitting analysis using the Prism software. (GraphPad Software).

### Clonogenic assay

PDAC cell lines were seeded in six-well plates at a density of 300 cells per well, cultured overnight, and treated with either 0.496 μM gemcitabine (PiSA Laboratories, Mexico), IC_50_-concentrations of C14, P8, or 5 μM deltarasin for 72 h. Subsequently, the medium was replaced with fresh supplemented medium every 3rd d for 10 d. Cells were fixed with 4% PFA at RT for 10 min and washed with distilled water. Cells were stained with 0.1% crystal violet in 0.1 M of citric acid for 30 min, washed with 1x PBS, dried, and photographed. To extract the dye for quantification, 14% acetic acid was applied for 20 min. The absorbance was then measured photometrically at 500 nm using a TECAN Fluorometer Infinite F500 (Tecan Austria GmbH).

### Apoptosis assay

5 × 10^5^ cells were seeded in six-well plates and cultured for 24 h. Then, cells were treated with an IC_50_ concentration of C14 and P8 for 24 h. Cells were detached with 0.25% trypsin, washed with PBS, and collected by centrifugation. Apoptosis was determined using the apoptosis/necrosis detection kit (catalog No. ab176749; Abcam) according to the manufacturer’s instructions and analyzed by flow cytometry on an FACS Calibur (BD Biosciences). Data were analyzed using FlowJo software 8.7.1 (Autissier et al, 2010).

### Ras activation assay

For the evaluation of Ras activation levels after treatment with deltarasin, gemcitabine, C14 or P8, a RAS-GTP pull-down assay was performed using the Ras Activation Assay Biochem Kit according to the standard procedure (BK008; Cystoskeleton, Inc.). Briefly, 3 × 10^6^ cells were lysed in ice-cold lysis buffer (400 μl) supplemented with complete EDTA-free Ultra Protease Inhibitor Cocktail and 1xPhosSTOP (Sigma-Aldrich). Lysates were centrifuged, the supernatants collected, and the protein concentration determined using the Pierce BCA Protein Assay kit (Thermo Fisher Scientific). 300 μg of total protein were incubated by end-over-end rotation with 100 μg of Raf-RBD–conjugated beads for 1 h, followed by centrifugation at 18.8*g* for 10 min. The beads were centrifuged at 18.8*g* for 10 min, rinsed with 500 liters of wash buffer, boiled in 2x Laemmli sample buffer, and then subjected to Western blot analysis using the pan-Ras and KRas (F234 Santa Cruz Biotechnology 1:100) antibody.

### Western blot

Cells were serum-starved for 16 h and pretreated with IC_50_ concentrations of C14, P8, gemcitabine, and deltarasin for 3 h. After pretreatment, cells were stimulated with 100 ng/ml epidermal growth factor for 10 min. Whole-cell extracts were obtained in lysis buffer (20 mM Tris–HCl [pH 7.5], 1 mM EDTA, 150 mM NaCl, 1% Triton X-100, 1 mM NaVO3, 1 mM NaF, 10 mM β-glycerophosphate, 1 mM phenylmethylsulfonyl fluoride, and 1.2 mg/ml complete Lysis-M [Roche] protease inhibitor cocktail). The protein extracts were forced through a 22-gauge needle 10 times and centrifuged for 10 min at 18.8*g* at 4°C, and the protein concentration was determined using the Pierce BCA Protein Assay kit (Thermo Fisher Scientific). Weighed tissue samples were snap-frozen, and then crushed in a mortar with liquid nitrogen. ProteoJETTM Mammalian Cell Lysis Reagent was used to lyze the samples after they had been transferred to a microcentrifuge tube (Thermo Fisher Scientific). Protein concentrations were measured after 15 min of spinning at 20,000*g*. SDS–PAGE was performed using 30 μg of protein per sample. Proteins were transferred onto PVDF membranes (Merck Millipore) and blocked for 1 h at RT using PBS containing 5% skim milk. Membranes were then incubated overnight at 4°C with the following primary antibodies: total ERK (1: 1,000; Cell Signaling-9102), p-ERK (1: 1,000; Cell Signaling-9101), total AKT (1: 1,000; Cell Signaling-9272), p-AKT (1: 1,000; Cell Signaling-4060), and anti-GAPDH as a loading control (1:100,000; Gene Tex-GTX100118). Immunodetection was performed using a ChemiDocTM Imaging System (Bio-Rad). Densitometry analysis was performed using ImageJ version 1.45 (National Institute of Health, USA).

### Treatment of subcutaneous pancreatic carcinoma xenograft

Immune-deficient NU/NU nude mice were maintained in pathogen-free conditions and fed with irradiated chow (CINVESTAV). 6-wk-old male mice were subcutaneously injected in the back with 5 × 10^6^ MIA PaCa-2 cells per tumor in 0.2 ml DMEM high-glucose medium containing 20% of matrigel. When MIA PaCa-2 cells reached palpable tumors (>150 mm^3^), mice were divided randomly into groups receiving intra-peritoneal injections once a day for 15 d of either vehicle (10% DMSO, 0.05% carboxy methyl cellulose in PBS), gemcitabine 40 mg/kg, C14 or P8 alone at 60, 30, 10, and 5 mg/kg, or the combination of C14/P8 30 mg/kg. Body weight and tumor sizes were measured daily. Tumor size was calculated using the following formula: ([length × width^2^]/2) in mm.

### Hematoxylin eosin stainings

1 d after the last treatment, the mice were euthanized using a CO_2_ chamber, and the xenograft tumors were resected, fixed in 4% buffered formalin, and embedded in paraffin. The tumors were cut using a microtome into 2-μm slices. For hematoxylin and eosin staining, the tissues were deparaffinized in xylene, hydrated in alcohol starting from absolute ethanol to distilled water, and stained for 2 min with Harris hematoxylin. Then, they were decolorized with 0.5% acidic alcohol and fixed in lithium carbonate for 1 min, washed in distilled water, then in 96% ethanol, stained with eosin (Sigma-Aldrich), washed and dehydrated in gradual alcohol series, allowed to dry at room temperature, mounted, and observed on a CX31, OLYMPUS microscope.

### Immunohistochemical staining of xenograft tumors

For immunohistochemical staining, tissues were deparaffinized in xylene and hydrated in depleted alcohols starting from absolute ethanol to distilled water. Subsequently, the epitopes were unmasked with 10 mM citrate buffer pH 6.03 in the Tender Cocker and washed with PBS pH 7.4. Endogenous peroxidases were blocked with 0.9% H_2_O_2_ for 15 min and 3% BSA for 1 h. The antibodies Ki-67 (API 3156 AA; BIOCARE MEDICAL), CK 19 (GeneTex, Inc), and CA125 (Biocare Medical) were diluted in 1x PBS containing 1% BSA, and incubated at room temperature for 40 min, washed with PBS for 3 min, incubated with the biotinylated secondary antibodies for 20 min at RT, washed with PBS for 3 min, incubated with streptavidin for 15 min, and washed again with PBS for 3 min. Finally, reactions were revealed using 4% DAB, counterstained with Harris hematoxylin for 30 s, washed with distilled water, and dehydrated in gradual changes of ethanol from distilled water to absolute ethanol, allowed to dry at RT, mounted, and observed on a CX31 OLYMPUS microscope.

### Primary pancreatic cancer biopsies from patients with PDAC

Pancreatic cancer tissues were provided by the Regional Hospital *1 de Octubre* of the “Institute of Security and Social Services for State Workers” (Project 002.2015; ISSSTE). The ethics committees of this hospital and CINVESTAV have approved all experimental procedures. Biopsies were collected in the hospital’s operating room. The tissue was cut to obtain 3-mm^3^ fragments, which were placed in six-well plates with 17% of glucose DMEM , 80% FBS, and 3% antibiotic, until cells from the tumor adhered to the plate and started forming monolayers. The serum percentage was gradually decreased until the cells could survive with 10% serum and 1% antibiotic. Fresh surgical resections were placed on ice in DMEM base medium without FBS but with 5% antibiotic).

### Patient-derived subcutaneous xenograft model

Male immune-deficient NU/NU nude mice were maintained in pathogen-free conditions with irradiated chow at 6 wk of age. 6-wk-old male NU/NU mice were subcutaneously injected with 5 × 106 MGKRAS004 and MGKRAS005 primary culture cells in 0.2 ml of DMEM high-glucose media with 20% matrigel in the torsos of the animals. When tumors were palpable (>150 mm^3^), mice were divided randomly into different groups receiving intra-peritoneal injections once daily for 15 d of either vehicle (10% DMSO and 0.05% carboxy methyl cellulose in PBS), 40 mg/kg gemcitabine, C14 or P8 alone at 60, 30, 10, and 5 mg/kg, or the combination of C14/P8 at 30 mg/kg. Bodyweight and tumor sizes were measured once a day. Tumor size was calculated using the following formula: ([length × width^2^]/2) in mm.

### Orthotopic xenograft model in NU/NU mice

At 6 wk old, male immune-deficient NU/NU nude mice were anesthetized with xylazine and ketamine. The spleen was located, a 0.5-cm incision was made in the skin and peritoneum, and the spleen was moved, allowing visualization of the pancreas. Subsequently, 1 × 10^6^ MIA PaCa-2 cells were inoculated in 50 μl of serum-free and phenol red-free essential minimal medium directly into the pancreas. Organs were placed back, and the peritoneum and skin were sutured with a self-absorbing suture. Mice were divided randomly into different groups receiving vehicle (10% DMSO and 0.05% carboxymethyl cellulose in PBS), C14, and P8 at 60, or 30 mg/kg alone, the combination of C14/P8 at 30 mg/kg each, or 40 mg/kg gemcitabine administered by intraperitoneal injection once daily for 15 d. A complete autopsy was performed to obtain organs for further analysis.

### Cell immunofluorescence

Cells were cultured on glass coverslips in 24-well plates until reaching confluence. They were fixed with 4% PFA for 20 min at 37°C, then washed with 1x PBS, and permeabilized with methanol/acetone 1:1 or 0.2% Triton-X100 for 10 min in a humid chamber. Then, samples were washed, and autofluorescence was blocked with NH_4_Cl at 50 μM for 20 min at 37°C. Unspecific binding was blocked with 2% BSA for 30 min at 37°C, and samples were incubated with the primary antibodies overnight at 4°C or for 90 min at 37°C, washed, and subsequently incubated with secondary antibodies labeled with fluorochrome for 60 min at 37°C, washed, and mounted in Vectashield containing DAPI. Images were taken using a confocal microscope (Leica SP8).

### Tissue immunofluorescence

Tissue cross-sections were deparaffinized in xylene and hydrated in degrading alcohols starting with absolute ethanol to distilled water. The epitopes were unmasked with 10 mM citrate buffer pH 6.03 in the Tender Cocker and washed with PBS pH 7.4. Endogenous peroxidases were blocked with 0.9% H_2_O_2_ for 5 min followed by 0.05 M NH4CL for 30 min at 37°C to decrease auto-fluorescence and washed with PBS-T three times. The primary antibodies (Table S1) were diluted in 1X PBS containing 1% BSA to block unspecific binding and incubated at RT for 60 min, washed with PBS for 3 min, incubated with secondary antibodies labeled with fluorochrome for 40 min at RT, and washed again with PBS for 3 min. Nuclei were labeled with DAPI, and images were taken using a confocal microscope (Leica SP8).

### DNA extraction

Genomic DNA was extracted from frozen human samples diagnosed with pancreatic cancer (MGKRAS003, MGKRAS004, and MGKRAS005) using the GenElute Mammalian Genomic DNA Miniprep Kit (G1N70; Sigma-Aldrich) according to the manufacturer’s instructions.

### PCR and sequencing

PCR was performed with ∼60 ng of genomic DNA using the forward and reverse primers at a concentration of 10 pmol. Forward: RASA1 5′-AAGGCCTGCTGAAAATGAC-3′ Reverse: RASA2 5′-TGGTCCTGCACCAGTAATATG-3′. PCR was performed in a TC-512 thermal cycler TECHNE for 20 cycles with an initial annealing temperature of 65°C, decreasing 0.5°C per cycle, followed by 15 cycles at 55°C annealing temperature. The PCR products were purified using the QIAprep Miniprep QIAGEN Kit. The purified PCR products were sequenced in the reverse direction.

### Genotoxicity assay

Bone marrow micronuclei were measured to assess the potential genotoxic effects of C14 and P8 substances (MacGregor et al, 1987). The test compounds were intraperitoneally administered once at a concentration of 40 mg/kg of gemcitabine, 60 mg/kg of C14 and P8, and 30 mg/kg of each C14 and P8 combined. Vehicle-treated and untreated mice served as control. Bone marrow cells were obtained 24 h and 15 d after treatment and stained with Giemsa-Wright (Diff-Quick; Harleco). 2,000 polychromatic erythrocytes were counted per animal using a bright-field microscope at 100x magnification to determine the number of micronucleated polychromatic erythrocytes.

### Toxicity assay

Toxicity of compounds C14 and P8 was determined using BALB/c mice treated intraperitoneally once daily for 15 d with 40 mg/kg gemcitabine, 60 mg/kg C14 and P8 alone, and the combination of C14/P8 at 30 mg/kg each. Vehicle-treated and untreated mice served as controls. Organs, blood, and urine were collected and analyzed for clinical parameters. Blood chemistry was performed using the Cobas c111 device (Roche) and hematic biometry using the Xp300 device (Sysmex).

### Statistical analysis

Statistical comparisons were performed by one-way analysis of variance (ANOVA) followed by Dunnett’s multiple comparisons test using GraphPad Prism version 8.0.0 for Windows (GraphPad Software). The data are shown as mean±SEM. A *P*-value of < 0.05 was considered statistically significant.

## Data Availability

Datasets used or analyzed in this study are available upon request to the corresponding author.

### Ethics statement

Ethical approval and consent to participate were obtained. The Institutional Animal Care and Use Committee (IACUC) of CINVESTAV, the regulatory office for approving research protocols involving laboratory animals, approved all experiments with mice (protocol number: 0201-16).

## Supplementary Material

Reviewer comments
