# Supplementary material for: Synergistic effect of antagonists to KRas4B/PDE6 molecular complex in pancreatic cancer
Source: Life Sci Alliance. 2023 Oct 9;6(12):e202302019. doi: 10.26508/lsa.202302019 (PMC10561825; doi:10.26508/lsa.202302019)
Supplement: Supplementary file 1 [file LSA-2023-02019_TableS1.docx]

**Table S1**. Interactions of compound C14 and P8 on the K-Ras4B/PDE6δ complex. Results obtained from the virtual selection analysis in heterodimeric crystallographic complex.

| **Ligand** | **P8** | **C14** |
| --- | --- | --- |
| **Structure** | 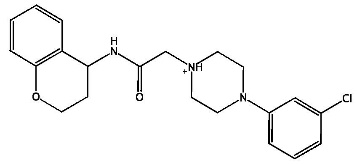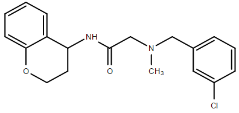 | 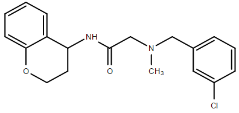 |
| **IUPAQ** | 2-[4-(3-chlorophenyl)piperazin-1-yl]-N-[(4R)-chroman-4-yl]acetamide | 2-[(3-chlorophenyl)methyl-methyl-amino]-N- chroman-4-yl-acetamide |
| **Formula/Molecular weight (g/mol)** | C_21_H_24_ClN_3_O_2_ /385,9 | C_19_H_21_ClN_2_O_2_/389.9 |
| **Docking score (kcal/mol)** | -15,7 | -14,7 |
| **Hydrogen Donor bond (kcal/mol)**  **KRas4B::Compound** | SER17(-3,88)  GLU37(-0,733)  ASP57(-0,867)  ALA59(-0,2)  GLU63(-0,1)  MET170(-0,422)  SER171(-0,6)  LYS175(-0,5)  LYS177(-0,2)  LYS178(-0,4)  SER181(-0,55) | GLN25 (-2.11)  GLU31 (-0.55)  ASP33 (-6.33)  GLU37 (-2.78)  ASP38 (-0.25) |
| **Hydrogen Acceptor bond (kcal/mol)**  **KRas4B::Compound** | SER17(-0,6)  ALA18(-3,1)  LYS171(-1,45)  GLY174(-1,6)  LYS175(-0,4)  LYS177(-0,4)  LYS178(-0,3) |  |
| **Ionic bond (kcal/mol)** | ASP57(-4,3) | ASP33 (-10.18) |
| **Pi bond (kcal/mol)**  **KRas4B::Compound** | SER17(-0,2)  ALA18(-0,55)  PHE28(-0,2)  GLU37(-0,2)  ALA59(-0,225)  MET170(-0,2)  LYS175(-0,3)  LYS177(-0,36) | ILE24 (-0.4)  TYR40 (-0.25)  GLU31 (-0.3)  GLU37 (-0.3)  ASP38 (-0.5) |
| **Pi bond (kcal/mol)**  **PDE6δ::Compound::** | PHE13(-5,0) |  |
| **Hydrogen bond (kcal/mol)**  **PDE6δ::CompounD** |  | GLU91 (-17.1) |
