# Supplementary material for: Synergistic effect of antagonists to KRas4B/PDE6 molecular complex in pancreatic cancer
Source: Life Sci Alliance. 2023 Oct 9;6(12):e202302019. doi: 10.26508/lsa.202302019 (PMC10561825; doi:10.26508/lsa.202302019)
Supplement: Supplementary file 2 [file LSA-2023-02019_TableS2.docx]

**Table S2**. Binding free energy components of protein-protein and protein-ligand complexes (in kcal/mol units).

| System | ΔE_vdw_ | ΔE_ele_ | ΔG_ele,sol_ | ΔG_npol,sol_ | ΔE_non-polar_ | ΔE_polar_ | DG_bind_ |
| --- | --- | --- | --- | --- | --- | --- | --- |
| Protein-protein | | | | | | | |
| KRASP4B-PDE-FAR | -123.08 (0.35) | -1546.94 (4.40) | 1608.17 (4.25) | -17.78 (0.04) | -140.86 | 61.23 | -79.63 (0.43) |
| KRASP4B_G12G_-PDE-C14 | -126.08 (0.49) | -1226.07 (4.83) | 1274.31 (4.60) | -18.76 (0.07) | -144.84 | 59.24 | -86.60 (0.62) |
| KRASP4B_G12C_-PDE-C14 | -119.58 (0.41) | -1609.83 (4.0) | 1646.28 (3.80) | -18.17 (0.05) | -137.75 | -36.45 | -100.30 (0.42) |
| KRASP4B_G12D_-PDE-C14 | -123.35 (0.60) | -1427.80 (8.0) | 1470.92 (7.60) | -18.75 (0.08) | -142.1 | -43.12 | -98.98 (0.80) |
| KRASP4B_G12V_-PDE-C14 | -113.35 (0.60) | -1572.80 (8.0) | 15870.92 (7.60) | -19.85 (0.08) | -133.84 | -33.84 | -99.36 (0.50) |
| KRASP4B_G12G_-PDE-P8 | -125.39 (0.35) | -1333.38 (5.12) | 1376.18 (4.80) | -19.18 (0.04) | -144.57 | 42.80 | -101.77 (0.59) |
| KRASP4B_G12D_-PDE-P8 | -126.16 (0.50) | -1326.34 (5.7) | 1368.18 (5.5) | -19.03 (0.05) | -145.19 | 41.84 | -103.35 (0.66) |
| KRASP4B_G12C_-PDE-P8 | -127.08 (0.37) | -1548.82 (3.8) | 1569.62 (3.6) | -19.86 (0.03) | -146.94 | 20.8 | -126.14 (0.50) |
| KRASP4B_G12V_-PDE-P8 | -124.17 (0.35) | -1339.84 (3.4) | 1367.63 (3.1) | -19.21 (0.03) | -143.38 | 27.79 | -115.59 (0.55) |
| KRASP4B_G12G_-PDE-C14/P8 | -145.24 (0.41) | -947.44 (3.60) | 1022.39 (3.74) | -20.24 (0.06) | -165.48 | 44.95 | -120.53 (0.65) |
| KRASP4B_G12D_-PDE-C14/P8 | -177.08 (0.37) | -1538.82 (3.3) | 1559.62 (3.6) | -19.52 (0.03) | -196.6 | 42.85 | -153.75  (0.40) |
| KRASP4B_G12C_-PDE-C14/P8 | -195.98 (0.37) | -1648.82 (3.5) | 1669.62 (3.3) | -18.16 (0.03) | -214.14 | 28 | -186.14  (0.42) |
| KRASP4B_G12V_-PDE-C14/P8 | -187.18 (0.42) | -1438.82 (4.8) | 1459.62 (4.6) | -19.6 (0.03) | -206.78 | 31.19 | -175.59  (0.52) |

Binding free energies and individual energy terms of complexes starting from docked conformations (kcal/mol). The polar (ΔEpolar = ΔEele + ΔGele,sol) and nonpolar

(ΔEnon-polar = ΔEvwd + ΔGnpol,sol) contributions are shown. All the energies are averaged over 500 snapshots at time intervals of 100 ps from the last 50

ns-long MD simulations and are in kcal/mol (± standard error of the mean)
