# Supplementary material for: Synergistic effect of antagonists to KRas4B/PDE6 molecular complex in pancreatic cancer
Source: Life Sci Alliance. 2023 Oct 9;6(12):e202302019. doi: 10.26508/lsa.202302019 (PMC10561825; doi:10.26508/lsa.202302019)
Supplement: Supplementary file 3 [file LSA-2023-02019_TableS3.docx]

**Table S3** Analogs to the leader compound C14 selected by means of bioinformatics programs.

| NUMBER | MOLECULE | logS | logP | ROTABLE LINK | FISA | ACCEPTOR | DONOR |
| --- | --- | --- | --- | --- | --- | --- | --- |
| 1 | 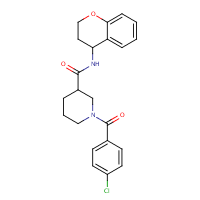 | -4 | 3,246 | 3 | 58,64 | 3 | 1 |
| 2 | 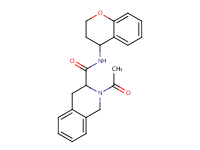 | -2 | 1,854 | 2 | 58,64 | 3 | 1 |
| 3 | 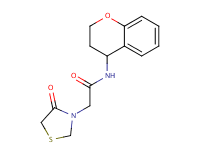 | -3.43 | 1,219 | 3 | 58,64 | 3 | 1 |
| 4 | 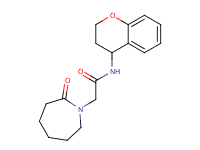 | -3 | 3,031 | 3 | 21,26 | 2 | 1 |
| 5 | 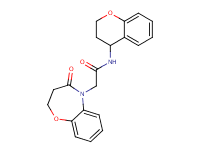 | -3 | 2,24 | 3 | 67,87 | 4 | 1 |
| 6 | 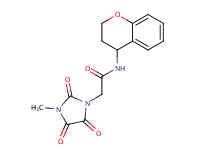 | -4 | 1,592 | 4 | 57,401 | 6 | 1 |
| 7 | 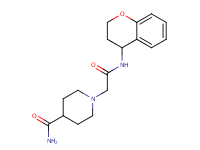 | -1.903 | -0,157 | 4 | 84,66 | 4 | 2 |
| 8 | 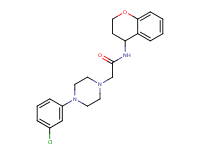 | -3 | 3,31 | 4 | 44,81 | 4 | 1 |
| 9 | 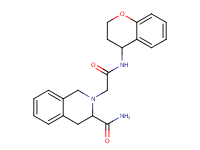 | -1 | 1,889 | 4 | 128,912 | 4 | 3 |
| 10 | 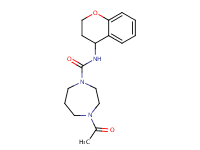 | -2 | 0,529 | 1 | 61,88 | 3 | 1 |
| 11 | 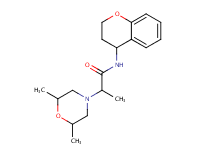 | -2 | 2,464 | 3 | 50,8 | 4 | 1 |
| 12 | 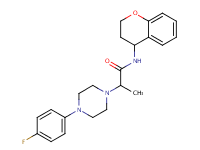 | -4 | 3,797 | 4 | 44,81 | 4 | 1 |
| 13 | 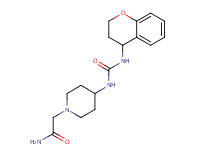 | -2.36 | 0,146 | 4 | 96,69 | 4 | 3 |
| 14 | 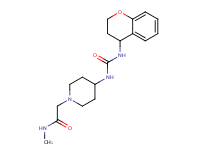 | -2.4 | 0,412 | 4 | 82,7 | 4 | 3 |
| 15 | 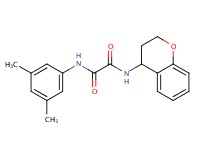 | -5 | 2,481 | 3 | 67,43 | 3 | 2 |
| 16 | 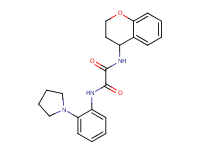 | -5 | 1,762 | 4 | 70,67 | 4 | 2 |
| 17 | 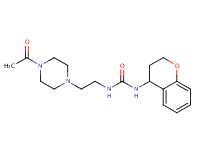 | -2.24 | 1,019 | 4 | 73,91 | 4 | 2 |
| 18 | 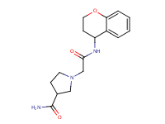 | -1.86 | 0,287 | 4 | 84,66 | 4 | 2 |
| 19 | 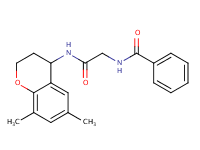 | -5 | 2,749 | 4 | 67,43 | 3 | 2 |
| 20 | 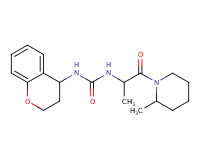 | -4 | 2,456 | 3 | 70,67 | 3 | 2 |
