# Supplementary material for: Synergistic effect of antagonists to KRas4B/PDE6 molecular complex in pancreatic cancer
Source: Life Sci Alliance. 2023 Oct 9;6(12):e202302019. doi: 10.26508/lsa.202302019 (PMC10561825; doi:10.26508/lsa.202302019)
Supplement: Supplementary file 5 [file LSA-2023-02019_TableS4.docx]

**Table S4** Characterization of biomarkers of origin and malignancy in primary cultures.

| **Immunofluorescence** | | | **MARKERS OF PANCREATIC ORIGIN** | | **MALIGNITY MARKER** | | | | | | | |  | |  | **Mutation in KRAS** | |
| --- | --- | --- | --- | --- | --- | --- | --- | --- | --- | --- | --- | --- | --- | --- | --- | --- | --- |
| **LINE CELLS** | | | **CK7** | **CK19** | **CEA** | **MUC1** | **MUC4** | **MUC16** | **EGFR** | **VIMENTIN** | **CYTOPLASMATIC**  **B-CATENIN** | **CYTOPLASMATIC E-CADHERINE** | **KI-67** | **STAGE** | |  |  |
| **PBDD33** | **WOMEN EPITHELIAL** | **PRIMARY CULTURE** | * | * | N | BASAL | N | N | BASAL | *** | N | N | *** | - | | ND |  |
| **JGCD28** | **MEN FIBROBLAST** | **PRIMARY CULTURE** | N | N | N | N | N | N | BASAL | *** | N | N | *** | - | | ND |  |
| **MGKRAS003** | **PANCREATIC CANCER** | **PRIMARY CULTURE** | *** | *** | *** | *** | *** | *** | *** | *** | *** | *** | *** | **G4** | | **G12G** |  |
| **MGKRAS004** | **PANCREATIC CANCER** | **PRIMARY CULTURE** | *** | *** | *** | *** | *** | *** | *** | *** | *** | *** | *** | **G4** | | **G12V** |  |
| **MGKRAS005** | **PANCREATIC CANCER** | **PRIMARY CULTURE** | *** | *** | *** | *** | ** | *** | *** | *** | *** | *** | *** | **G4** | | **G12C** |  |
