# Supplementary material for: Synergistic effect of antagonists to KRas4B/PDE6 molecular complex in pancreatic cancer
Source: Life Sci Alliance. 2023 Oct 9;6(12):e202302019. doi: 10.26508/lsa.202302019 (PMC10561825; doi:10.26508/lsa.202302019)
Supplement: Supplementary file 8 [file LSA-2023-02019_TableS5.docx]

**Table S5**. Side effects obtained in BALB/c mice treated with Gemcitabine, C14, P8 and C14/P8

|  | GEMCITABINE | C14 | P8 | C14/P8 |
| --- | --- | --- | --- | --- |
| DIARRHEA | YES | NO | NO | NO |
| RECTAL PROLAPSE | YES | NO | NO | NO |
| INTESTINAL TORSION SYNDROME | YES | NO | NO | NO |
| NEUTROPENIA | G2 | NO | NO | NO |
| LEUKOPENIA | G2 | NO | NO | NO |
| MUSCLE DECLINE | YES | NO | NO | NO |
| LACK OF APPETITE | YES | NO | NO | NO |
| WEIGHTLOSS | YES | NO | NO | NO |
| HEPATIC ENZYMES | G1 | NORMAL | NORMAL | NORMAL |
| GUE | | | | |
| GLUCOSE | 500 mg/dl | NOT DETECTED | NOT DETECTED | NOT DETECTED |
| PROTEIN | 2000 mg/dl | 10 mg/dl | 10 mg/dl | 10 mg/dl |
| BILIRUBIN | 70 mg/dl | NOT DETECTED | NOT DETECTED | NOT DETECTED |
