# Supplementary material for: Synergistic effect of antagonists to KRas4B/PDE6 molecular complex in pancreatic cancer
Source: Life Sci Alliance. 2023 Oct 9;6(12):e202302019. doi: 10.26508/lsa.202302019 (PMC10561825; doi:10.26508/lsa.202302019)
Supplement: Supplementary file 9 [file LSA-2023-02019_TableS6.docx]

**Table S6** Side effects presented in patients treated with standard chemotherapy in Mexico

| GEMCITABINE | FOLFIRINOX/  CAPECITABINE | FOLFIRINOX/  CAPECITABINE/ OXALIPLATINO | WEIGHT START | FINAL WEIGHT | START HEIGHT | FINAL HEIGHT |
| --- | --- | --- | --- | --- | --- | --- |
| ASTENIA G1 | FATIGUE G1 | BONE PAIN | 60 Kg | 40 Kg | 1.57 | 1.5 |
| NAUSEAS G1 | HYPOREXIA G1 | FATIGUE G2 |  |  |  |  |
| DIARRHEA G1 | NAUSEAS G1 | SICKNESS |  |  |  |  |
| EPIGASTRALGIA | ECOG PALPABLE | HYPOREXIA |  |  |  |  |
| FATIGUE G1 | NEUTROPENIA G2 | ECOG PALPABLE |  |  |  |  |
| HYPOREXIA G1 | ABNORMAL BH | QS NORMAL LIMITS |  |  |  |  |
| ECOG WITHOUT PALPABLE ALTERATIONS | PLATELET INCREASE | ABNORMAL BH |  |  |  |  |
| BH RECOVERED | LEUKOCYTIC DECREASE | HEPATIC ENZYMES IN G1 |  |  |  |  |
| HEPATIC ENZYMES G1 | ECOG PALPABLE | PRESENTS LOW |  |  |  |  |
|  | PRESENTS LOW MEDULAR RESERVE | MEDULAR RESERVE |  |  |  |  |
